# Elimination of Reference Mapping Bias Reveals Robust Immune Related Allele-Specific Expression in Crossbred Sheep

**DOI:** 10.3389/fgene.2019.00863

**Published:** 2019-09-19

**Authors:** Mazdak Salavati, Stephen J. Bush, Sergio Palma-Vera, Mary E. B. McCulloch, David A. Hume, Emily L. Clark

**Affiliations:** ^1^The Roslin Institute and Royal (Dick) School of Veterinary Studies, University of Edinburgh, Easter Bush, Edinburgh, United Kingdom; ^2^Leibniz Institute for Farm Animal Biology (FBN), Institute for Reproductive Biology, Dummerstorf, Germany; ^3^Mater Research Institute-University of Queensland, Translational Research Institute, Woolloongabba, QLD, Australia

**Keywords:** allele-specific expression, mapping bias, RNA-Seq, sheep, transcriptome, WASP, GeneiASE

## Abstract

Pervasive allelic variation at both gene and single nucleotide level (SNV) between individuals is commonly associated with complex traits in humans and animals. Allele-specific expression (ASE) analysis, using RNA-Seq, can provide a detailed annotation of allelic imbalance and infer the existence of cis-acting transcriptional regulation. However, variant detection in RNA-Seq data is compromised by biased mapping of reads to the reference DNA sequence. In this manuscript, we describe an unbiased standardized computational pipeline for allele-specific expression analysis using RNA-Seq data, which we have adapted and developed using tools available under open license. The analysis pipeline we present is designed to minimize reference bias while providing accurate profiling of allele-specific expression across tissues and cell types. Using this methodology, we were able to profile pervasive allelic imbalance across tissues and cell types, at both the gene and SNV level, in Texel×Scottish Blackface sheep, using the sheep gene expression atlas data set. ASE profiles were pervasive in each sheep and across all tissue types investigated. However, ASE profiles shared across tissues were limited, and instead, they tended to be highly tissue-specific. These tissue-specific ASE profiles may underlie the expression of economically important traits and could be utilized as weighted SNVs, for example, to improve the accuracy of genomic selection in breeding programs for sheep. An additional benefit of the pipeline is that it does not require parental genotypes and can therefore be applied to other RNA-Seq data sets for livestock, including those available on the Functional Annotation of Animal Genomes (FAANG) data portal. This study is the first global characterization of moderate to extreme ASE in tissues and cell types from sheep. We have applied a robust methodology for ASE profiling to provide both a novel analysis of the multi-dimensional sheep gene expression atlas data set and a foundation for identifying the regulatory and expressed elements of the genome that are driving complex traits in livestock.

## Introduction

Allele-specific expression (ASE) is the imbalance of allelic expression between parental (diploid) copies at the same locus (Barlow and Bartolomei, 2014). It is most commonly associated with *cis*-acting regulatory variation that may mediate parent-of-origin, sex- or tissue-specific transcription of one allele relative to the other (Renfree et al., 2009; Hasin-Brumshtein et al., 2014). In a single individual, where there are informative sequence variants (i.e., heterozygote loci) that distinguish the products of two alleles, ASE can be detected by RNA sequencing (Chamberlain et al., 2015; GTEx Consortium et al., 2017; Cao et al., 2019; Guillocheau et al., 2019). The ratio of allelic read counts obtained from RNA-Seq data sets can be used as a reliable proxy for ASE [i.e., *ASEratio* = *CountsAllele*1/(*CountsAllele*1 + *CountsAllele*2)] (Edsgärd et al., 2016).

Large and complex RNA-Seq data sets give rise to unique and interesting computational challenges, in particular the elimination of reference mapping bias in ASE analysis of diploid genomes. RNA-Seq data are commonly mapped against reference genomes which are typically “flat,” with each position represented only by the reference (most abundant) allele. As such, reads containing heterozygous loci are more likely to be erroneously mapped (Degner et al., 2009; Stevenson et al., 2013; Hodgkinson et al., 2016). This can lead to high false-positive ASE locus discovery rates (Degner et al., 2009). Although development of *de novo* transcript assemblers (Zerbino and Birney, 2008), usage of personalized reference genomes (Rozowsky et al., 2011; Smith et al., 2013), variant-aware aligners (Xin et al., 2013; Hach et al., 2014), and mapping-free quantification e.g., Kallisto (Bray et al., 2016) have resolved some of these issues, reference allele mapping bias remains a considerable challenge in ASE studies. In the absence of “trios” of animals or reference population phased haplotype information, which are rare for livestock, correction of mapping bias *via* synthetic reads with either N masking or alternative mapping bias correction at the heterozygote sites, has proven a robust alternative for ASE discovery (Degner et al., 2009; Mayba et al., 2014; van de Geijn et al., 2015; Miao et al., 2018). In 2015, Van de Geijn et al. benchmarked the WASP software mapping correction strategy against N-masked reads and personal genome mapping. WASP showed consistent correct mapping of reads with multiple alleles and lower false discovery rates (FDR) in comparison to the other two methods (van de Geijn et al., 2015). The analysis pipeline we present in this manuscript is based on WASP’s methodology and is designed to minimize reference bias while providing accurate profiling of allele-specific expression in large and complex RNA-Seq data sets.

We have developed an ASE analysis pipeline using the combination of software available under open license, WASP (reference mapping bias removal) (van de Geijn et al., 2015), GATK (ASEReadCounter) (McKenna et al., 2010; Van der Auwera et al., 2013), and GeneiASE (Liptak-Stouffer aggregative ASE gene model) (Edsgärd et al., 2016). The GeneiASE model is capable of testing ASE at the gene level using two approaches: i) static ASE, which measures allelic imbalance within a gene (i.e., when ASE variants are located within the boundaries of the gene); and ii) individual condition-dependent ASE (ICD), which measures inducible ASE in a gene under an environmental pressure between two timepoints (i.e., in stimulated or unstimulated immune cells).

In addition to ASE at the gene level, we can also measure significant ASE at the single-nucleotide level (SNV). ASE has been shown to be enriched within expression quantitative trait loci (eQTL) regions (Montgomery et al., 2010); therefore, identifying ASE variants can be useful for understanding the transcriptomic control of complex traits in livestock. Complex trait mapping of ASE loci has been associated with phenotypes, such as resistance to Marek’s disease in chicken (Meydan et al., 2011) and pigmentation patterns in sheep (García-Gámez et al., 2011).

Understanding ASE is also important because cross-breeding now underlies most livestock production systems. Knowledge of ASE may provide insights into the molecular basis of the complex phenomenon of hybrid vigor, as emphasized by recent studies on two Chinese goat breeds and their F1 hybrids (Cao et al., 2019) and in F1 crosses of two highly inbred chicken lines (Zhuo et al., 2017). In this study, we measure ASE in crossbred sheep. Sheep are an economically important livestock species in many countries across the globe and particularly in emerging economies. The identification of prevalent ASE in populations or breeds, especially in economically relevant phenotypes and tissues could be used to improve genomic prediction in sheep breeding programs, such as those that have been established in Australia and New Zealand (Daetwyler et al., 2010).

Using the methodology we describe, for mapping bias correction and robust positive ASE discovery, we were able to profile pervasive allelic imbalance across tissues and cell types, at both the gene and SNV level, in Texel×Scottish Blackface sheep. We analyzed a subset of total RNA-Seq libraries from liver, spleen, ileum, thymus, and bone marrow-derived macrophages (BMDM) (±) lipopolysaccharide (LPS) from six individual adult crossbred sheep to produce a detailed picture of allelic imbalance in immune-related tissues and cell types. We chose to focus this analysis on immune-related tissues in part because of the depth of available sequence in those tissues, and in part because they contain abundant immune cell populations. The diversity of cell populations is reflected in the transcriptional complexity of immune tissues and cell types in the sheep gene expression atlas data set (Clark et al., 2017; Bush et al., 2019). As such, this subset of tissues gave us a transcriptionally rich data set in which to measure ASE. We also included BMDMs stimulated and unstimulated with LPS to mimic infection with Gram-negative bacteria to test whether ASE changed in response to stimulation with LPS in these cells. By measuring ASE in these tissues and cell types from sheep we were able to: i) provide insight into how pervasive ASE is across tissues at the gene and SNV level, ii) generate tissue-specific ASE profiles, iii) investigate sex-specific patterns of ASE, and iv) determine the extent to which ASE changes in response to stimulation with LPS in an immune cell type. This novel analysis of the multi-dimensional sheep gene expression atlas data set provides a foundation for further analysis of the regulatory and expressed elements of the genome that are driving complex traits in sheep.

## Methods

### Sample Preparation and RNA Extraction

Data from three male and three female Texel×Scottish Blackface (T×BF) sheep from the sheep gene expression atlas project (Clark et al., 2017) were used in this study. The data set including: one cell type (BMDMs (±) LPS treatment) and four tissues (thymus, spleen, liver and ileum). Tissue collection, storage, and RNA extraction are described in Clark et al. (2017). BMDMs were cultured *in vitro* for 7 days in the presence of macrophage colony-stimulating factor (CSF1 (10^4^ U/ml)) and unstimulated (0 h −LPS) and stimulated (7 h +100 ng/ml LPS) samples of BMDMs were obtained as previously described (Clark et al., 2017). A total of two samples (one thymus and one spleen) did not pass the RNA quality control (RNA integrity number (Mueller et al., 2004); RIN^e^ >7) and were not included in the sheep gene expression atlas. Library preparation was performed by Edinburgh Genomics (Edinburgh Genomics, Edinburgh, UK). All total RNA Illumina TruSeq libraries (125 bp paired end) were sequenced at a depth of > 100 million reads per sample.

### Reference Mapping Bias Removal

BAM files from RNA-Seq data were previously produced by mapping fastq files to the Oar v3.1 top level DNA fasta track, using HISAT2 (default mismatch penalty MX = 6 MN = 2) as previously described (Clark et al., 2017). Detailed settings and parameters for all the tools used to generate the BAM files can be found at FAANG (2018). These BAM files were used to locate reads with heterozygote loci using WASP’s find_intersecting.py script (van de Geijn et al., 2015). The intersection of reads and heterozygote loci in all samples were based on the Ensembl v92 variant call format (VCF) track (Ensembl v92: ovis_aries_incl_consequences.vcf.gz). Briefly, the Ensembl VCF file was filtered for bi-allelic variants within exonic regions, 5k up or downstream of exonic regions (5′ or 3′ UTRs) and intronic regions of all transcripts within the Oar3.1 sheep assembly (exclusion of indels and intergenic variants). These variants were used in WASP’s find_intersecting.py script to extract reads mapped to coordinates containing variants for each gene. As a result, reads aligned to exonic, 5′ or 3′ UTRs and intronic regions were separated into reads intersecting heterozygote loci and reads that did not intersect heterozygote loci. Synthetic copies of reads intersecting heterozygote loci were created with the alternate allele flipped to the remaining options of A, T, C, or G [up to 6 loci/read(2^n^) max 64 combinations of synthetic reads] using parameters defined in WASP (van de Geijn et al., 2015). This was followed by remapping of the synthetic reads using HISAT2 (default mismatch penalty MX = 6 MN = 2) (Li and Durbin, 2009; Kim et al., 2015) and eliminating the original reads (and their synthetic copies) which mapped to a different coordinate in any of its synthetic copies (WASP’s filter_reads.py) (van de Geijn et al., 2015). After merging the retained reads with that did not intersect heterozygote loci, a final BAM file was produced for ASE read counting step (WASP’s remove_dup.py).

### Allelic Read Counts and Depth Filtration

**Figure 1:**
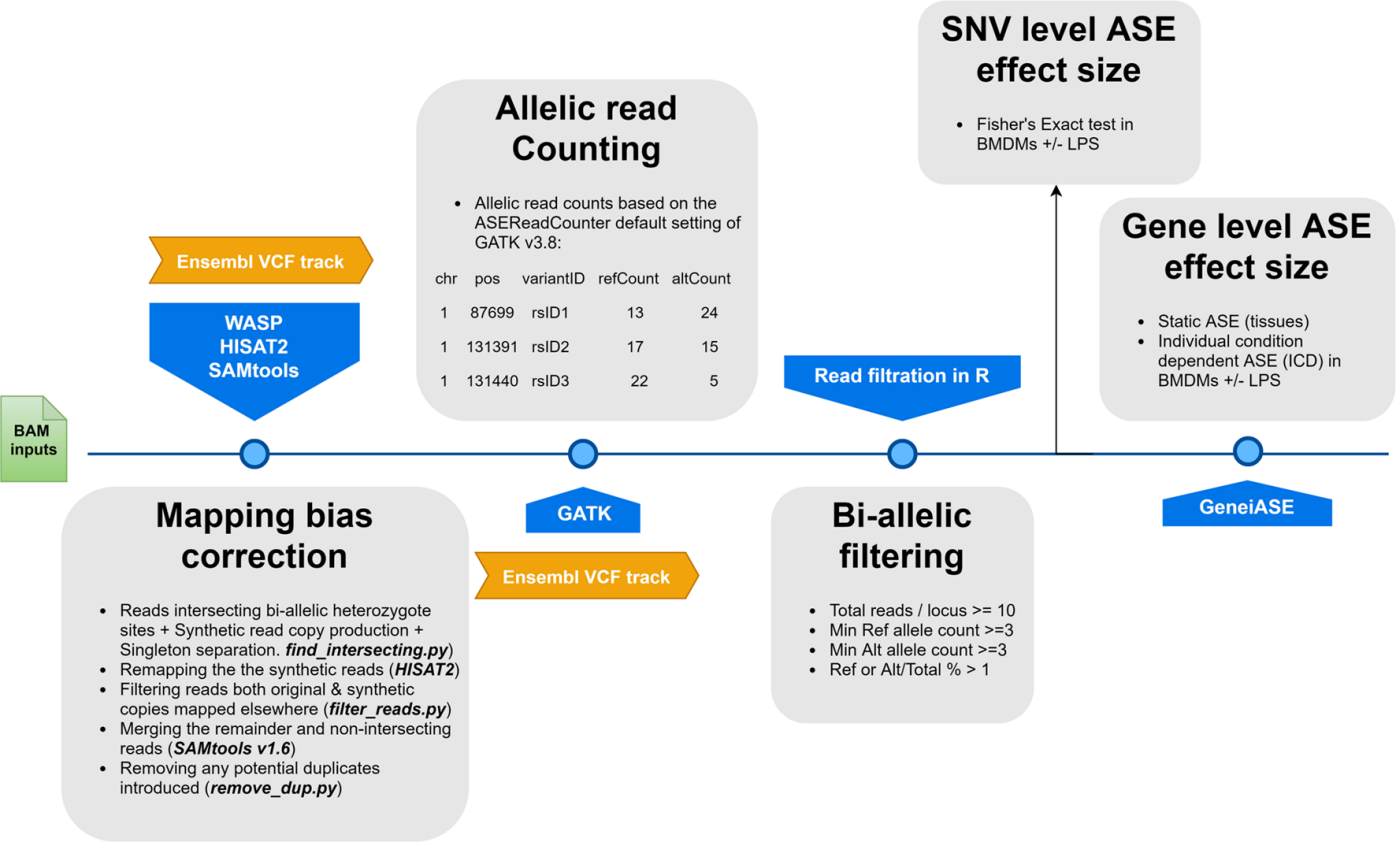
A flowchart of the allele-specific expression analysis pipeline applied to the sheep gene expression atlas data set and optimized for WASP and GeneiASE programs. The remapping was carried out using HISAT2 (Kim et al., 2015) in combination with SAMtools (Li et al., 2009). The Genome Analysis Toolkit v 3.8 was used for the ASE read counting section.

Allele-specific read counting was carried out using the ASEReadCounter module of GATK v3.8 with parameters -mmq 50 and -mbq 25 (McKenna et al., 2010). Multiple pre-processing steps were performed prior to GeneiASE input as instructed by Edsgärd et al. (2016), which included preparing per chromosome indices, merging the variant set with corresponding gene coordinates, and bi-allelic expression filtering. Loci with < 10 reads mapped were excluded, as were loci with < 3 reads, or < 1% of the total reads, mapped to both the reference and alternative allele. This form of filtration will eliminate loci exhibiting mono-allelic expression (MAE) as previously described (Degner et al., 2009; Stevenson et al., 2013; Mayba et al., 2014). Producing evidence of MAE using total RNA-Seq data sets produced by Illumina short read sequences without parent of origin genotypes or imprinting information has been a controversial issue (DeVeale et al., 2012). Our data set did not include the trios of animals or personalized genomes that would be necessary to resolve MAE. As such, we decided to exclude MAE altogether for our analysis using stringent bi-allelic filtration criteria. Similar bi-allelic filtration criteria have been previously used routinely in ASE studies (Mayba et al., 2014; Chen et al., 2016a; Edsgärd et al., 2016; GTEx Consortium et al., 2017; Raghupathy et al., 2018; Cao et al., 2019; Guillocheau et al., 2019; Gutierrez-Arcelus et al., 2019). The workflow of the analysis pipeline for ASE analysis is detailed in Figure 1.

### Experimental Design for Defining Allele-Specific Expression

ASE was defined according to the following three categories:

- Static ASE: which is inherent allelic imbalance (AI) in each gene calculated by ASE at all heterozygote loci (i.e., *ASE* = *Counts RefAllele*/(*Counts RefAllele* + *Counts AltAllele*)) within the boundaries of the gene. The effect size of ASE at gene level was produced by aggregation of the ASE effect size at SNVs within the gene boundaries (the Liptak-Stouffer method, applied by the GeneiASE aggregative model). A null distribution of ASE effect size for genes in each transcriptome was produced by random sub-sampling (n = 1 × 10^5^) from a pool of genes having min 2 and max 100 loci within their boundaries. The ASE effect size of each gene (aggregated using Liptak-Stouffer) was then tested against the null distribution of the same SNV number, *via* a modified bi-nomial test (2×1 table). Distribution of p values was examined for uniformity prior to FDR correction (**Supplementary Figures S1**, **S2**, and **S3**) (Benjamini and Hochberg, 1995; Pounds and Cheng, 2006; Barton et al., 2013).
- Individual condition-dependent ASE (ICD-ASE): in which the same ASE effect size was calculated for each gene in the treated versus the untreated timepoints of the same sample (i.e., BMDM ± LPS). The log2ratio (ASE_treated_/ASE_untreated_) was used in a beta-binomial test (2×2 table) similar to the static mode. The details of this aggregative model have been previously described in the GeneiASE publication (Edsgärd et al., 2016).
- Condition-dependent ASE at SNV level: in which a contingency table was produced for read counts (ref and alt) for every SNV, present both in treated and untreated conditions (BMDM ± LPS) (2×2 table) and a Fisher’s exact test performed followed by *p* value multiple testing correction (Benjamini and Hochberg, 1995). The *p* values from loci showing ASE and shared by the six adult sheep (ID and coordinate) were unified, using the Stouffer method (Dewey, 2016; Dewey, 2019) and presented as FDR for each locus.

Static ASE was calculated in both tissues and BMDMs (each timepoint was considered separately for BMDMs). Condition-dependent ASE analysis was carried out only in BMDMs ± LPS both at gene (ICD-ASE) and SNV (Fisher’s exact) level to study LPS-inducible ASE.

### Statistical Analysis and Thresholds Applied

The extraction, transformation and loading of the all data sets and subsequent statistical analysis was carried out in R version 3.4 or higher unless stated otherwise (R Core Team, 2017). System query language join statements (Wickham et al., 2019) were used to compare lists of ASE genes or SNVs between samples. Raw *p* values resulting from all three types of ASE analysis were corrected for multiple testing *via* Benjamini-Hochberg FDR calculations (Benjamini and Hochberg, 1995). The passing threshold of significance in all analyses was considered to be FDR < 0.1 (10%) except for the Fisher’s exact test association study. Genes showing ASE in multiple tissues were considered those for which four or more of the six sheep had significant ASE.

## Results

### Estimation of Heterozygous Sites Across All Individuals

**Figure 2:**
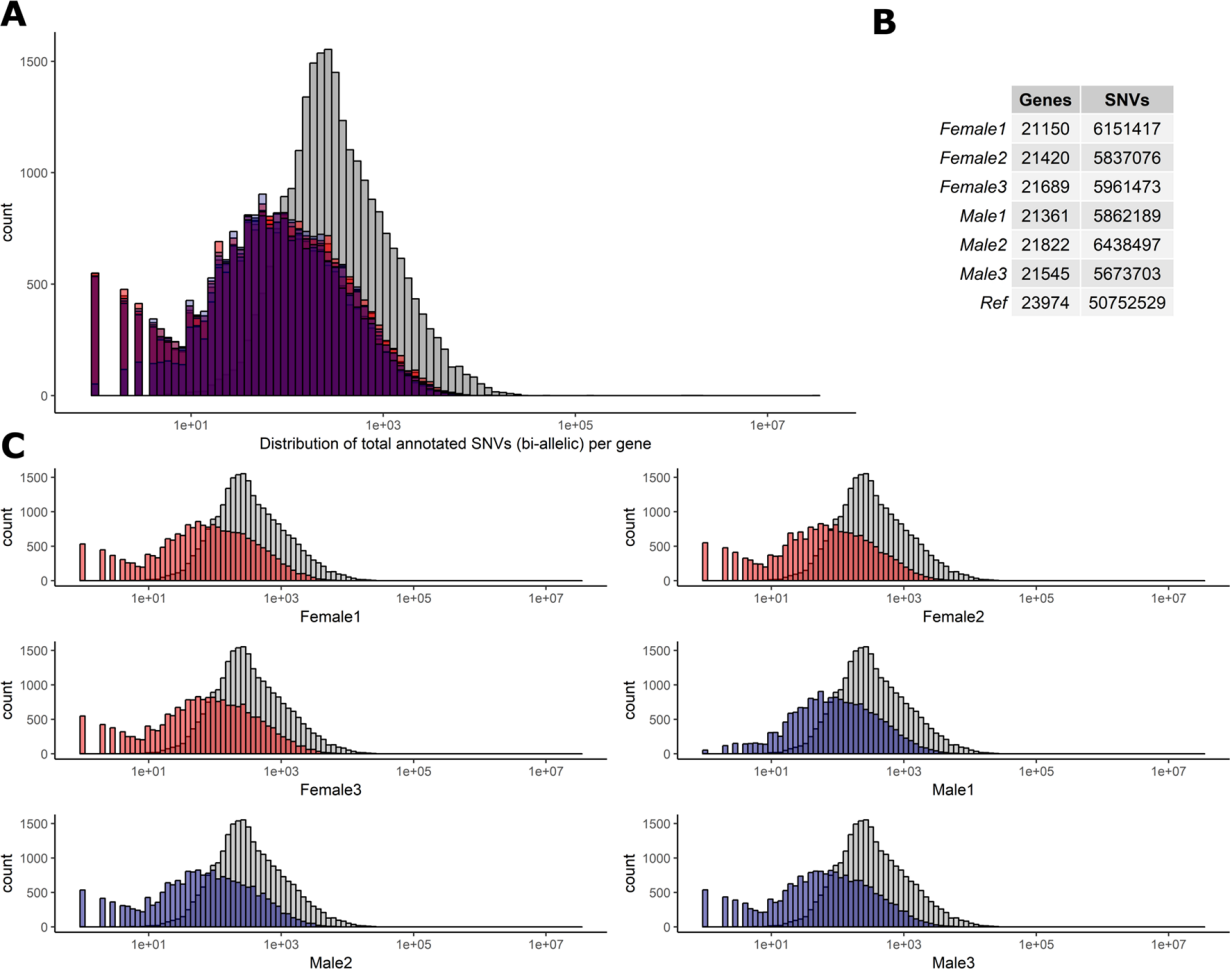
Distribution of biallelic SNVs expressed per gene in each of the six T×BF sheep. The total number of SNVs was averaged across thymus, liver, ileum, and spleen for every animal. Over 5×10^7^ SNVs were gathered using Ensembl v.92 VCF track. The total number of SNVs per genes is averaged across four tissue RNA-Seq in each animal (∼5.9 × 10^6^). **(A)** Histogram of SNVs per gene counts in the reference track (Ensembl in grey) and six sheep in red (females) and blue (males) overlaid. **(B)**. The overall numbers of genes and SNVs detected in each animal (averaged over four tissues). **(C)** Individual histograms from section A with females in red and males in blue.

To determine the level of heterozygosity present in the RNA-Seq data we first assessed the number of bi-allelic heterozygote sites per individual for each of the six sheep (range = 5,673,703–6,438,497) detailed in **Figure 2**. Individual variation was observed in the SNVs per gene in each sheep (**Figures 2A, C**). However, there was no significant difference in the total number of bi-allelic SNVs captured in the RNA-Seq data across all six individuals or between the male and female sheep included in the study (**Figure 2B**). The bi-allelic SNVs captured in the RNA-Seq data set were annotated using the Ensembl v.92 (Zerbino et al., 2018) reference VCF track. The distribution of SNVs per gene in the Ensembl track is tail-inflated in comparison to the RNA-Seq data **Figure 2A**. This issue could be due to erroneous assignment of SNVs in hypervariable and repetitive regions, multi-allelic SNVs or simply that there are variants in the Ensembl track that are not expressed (transcribed). The distribution of SNVs for each individual is shown in **Figure 2C**.

### Reference Mapping Bias Elimination and Quality Control

**Figure 3:**
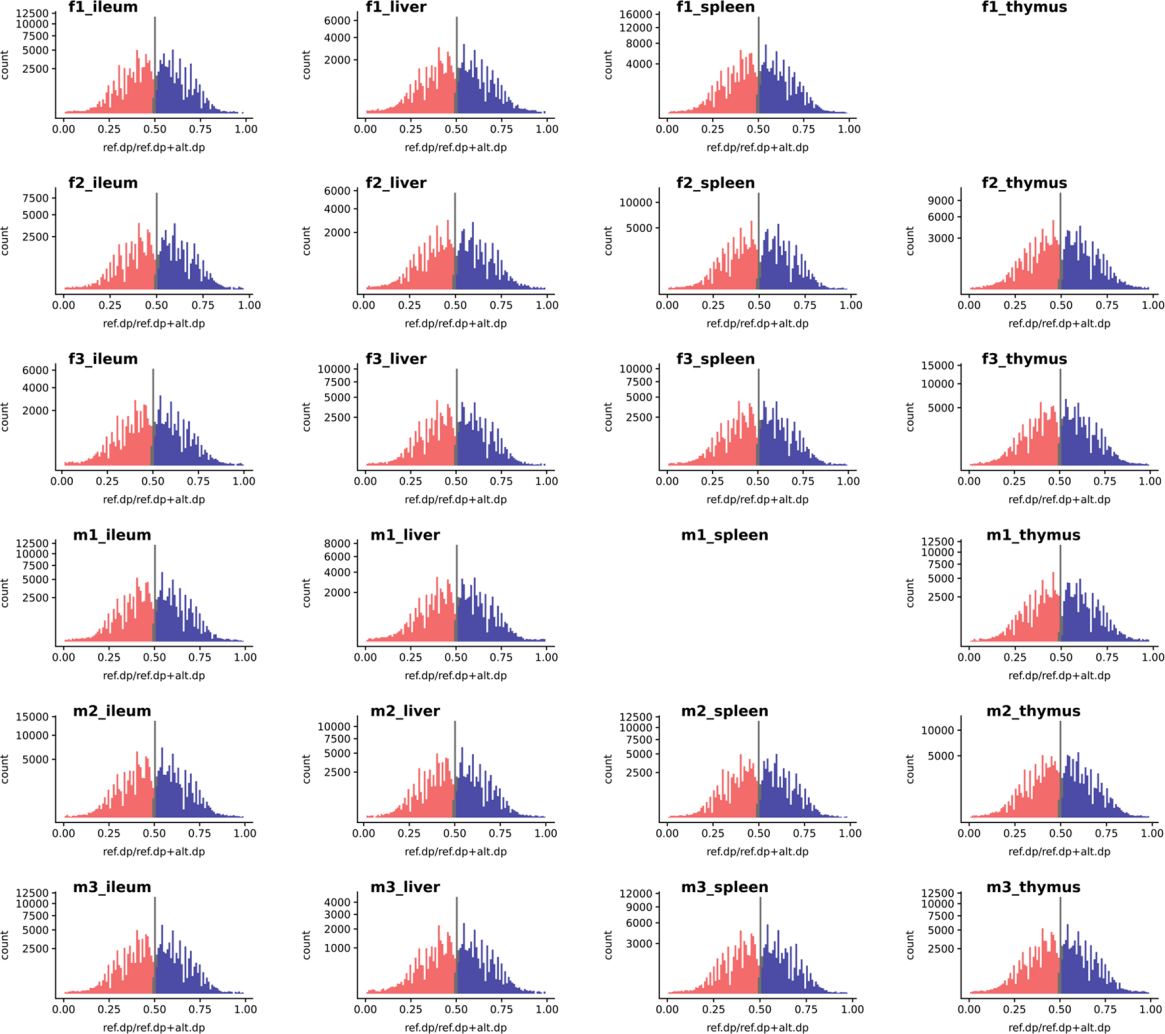
The histogram of a global reference allelic ratio at every locus in the tissues. The distribution of ref allelic ratio showed a balanced profile without any 0 or 1 inflation which is observed in the presence of reference mapping bias. The allelic ratio above 0.51 is shown in blue and below 0.49 in red while balanced bi-allelic expression (0.49–0.51) is colored in gray. Ref.dp, read counts for reference allele; Alt.dp, read counts for alternate allele. The y axis is square root scaled. As discussed in the text SNP that display MAE are not present in any of the samples analyzed, indicating there was no inflation in either 0 or 1 allelic ratio.

We used the WASP ref bias removal script to successfully minimize ref allele mapping bias in the RNA-Seq samples. The mapping bias was assessed by global distribution of the allelic ratio, i.e., ref_counts_/alt_counts_ + ref_counts_ in each RNA-Seq sample, as shown in **Figure 3** (WASP metrics are included in **Supplementary Figures S10**, **S11**, and **S12**). The ASE discovery rate at the SNV level, on average, constituted 5.8% of the heterozygote loci that passed the minimum filtration criteria in each individual (0.1% of the total expressed). This portion of the transcriptomic variants belonged to an average of 103 genes in each tissue transcriptome (approx. 1%) or 300 in each individual (**Supplementary Figures S5** and **S6**). As shown in **Supplementary Figure S6**, expression level varies across tissues but does not affect the distribution of ASE SNVs.

### Genes Exhibiting Tissue-Specific and Pervasive ASE Signatures

**Figure 4:**
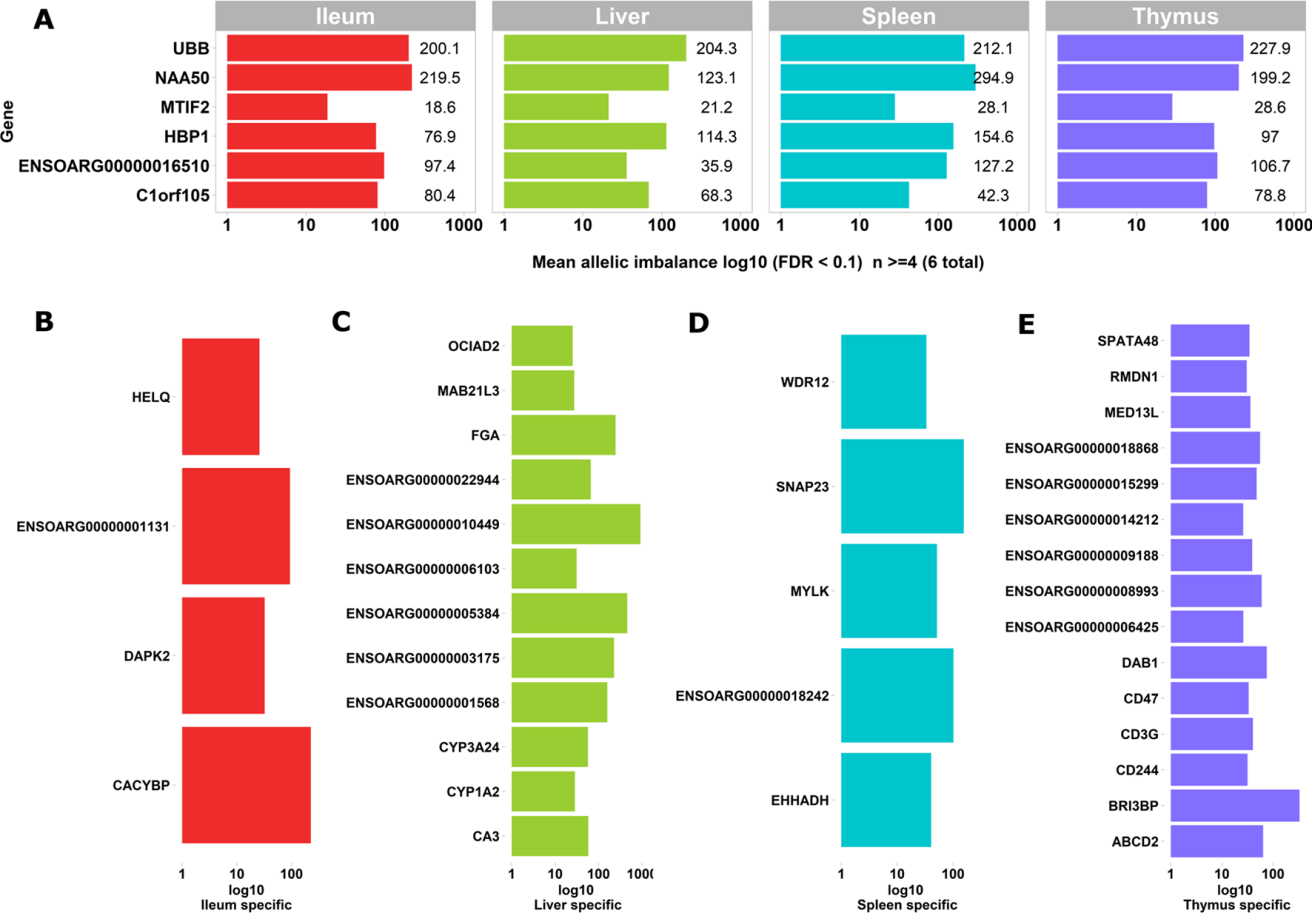
Genes exhibiting static ASE shared across tissues from all six sheep. The x axis represents the mean allelic imbalance (averaged static ASE across sheep in each tissue). **(A)** Genes shared by four tissues with significant (false discovery rate [FDR], < 0.1) static ASE. **(B)** ASE genes private to Ileum. **(C)** Private to liver. **(D)** Private to spleen. **(E)** Private to thymus.

We used the static mode of GeneiASE to investigate pervasive and tissue-specific ASE profiles across all of the available samples. Static ASE represents inherent allelic imbalance (AI) in each gene calculated by ASE at all heterozygote loci. The number of genes showing significant static ASE in immune-related tissues across the six sheep are summarized in **Table 1**. On average, approximately 0.5% of the genes in each tissue-specific transcriptome showed significant ASE (approx. 1% of the filtered set of genes). Pervasive ASE genes were investigated by applying the minimum 67% shared rule (i.e., an ASE gene was considered “shared” when it exhibited ASE in a minimum of four of six sheep). A list of ASE genes with significant allelic imbalance (AI) in all tissues, when the effect size was averaged across six sheep, was compiled (**Figure 4A**) (Static ASE measured by GeneiASE’s Liptak-Stouffer method). Six genes exhibited pervasive ASE across tissues (i.e., they were shared across all four tissues). In the order of allelic imbalance effect size they were *NAA50* (N(alpha)-acetyltransferase 50, NatE catalytic subunit) with highest ASE effect size in spleen, *UBB* (ubiquitin B) in thymus, *HBP1* (HMG-box transcription factor 1), and *ENSOARG00000016510* both in spleen, *C1orf105* (chromosome 1 open reading frame 105) in ileum and *MTIF2* (mitochondrial translational initiation factor 2) in thymus.

**Table 1:** Total number of genes with significant static ASE in proportion to genes containing informative SNVs (filtered). Total expressed: Average number of genes being expressed in all 4 tissues. Total filtered: Average number of genes (containing heterozygote loci) passing read bi-allelic filtration criteria in 4 tissues. Tissue break-down has been presented as count (%ASE/filtered).

Sheep	Thymus	Spleen	Liver	Ileum	Total filtered	Total expressed
Female 1	-	136 (1.31%)	153 (1.47%)	92 (0.88%)	10,379	21,150
Female 2	136 (1.28%)	70 (0.66%)	116 (1.09%)	70 (0.66%)	10,572	21,420
Female 3	151 (1.22%)	75 (0.60%)	105 (0.85%)	140 (1.13%)	12,326	21,689
Avg.	143 (1.25%)	94 (0.85%)	125 (1.13%)	101 (0.89%)	11,092	21,419
Male 1	95 (0.92%)	-	157 (1.52%)	86 (0.83%)	10,282	21,361
Male 2	125 (0.99%)	54 (0.43%)	86 (0.68%)	80 (0.63%)	12,514	21,822
Male 3	106 (1.01%)	71 (0.67%)	110 (1.04%)	82 (0.78%)	10,480	21,545
Avg.	109 (0.97%)	62 (0.55%)	118 (1.08%)	83 (0.74%)	11,092	21,576
Total avg.	126 (1.13%)	78 (0.70%)	121 (1.09%)	92 (0.82%)	11,092	21,497

Sets of genes with tissue-specific ASE profiles were also captured (**Figures 4B–E**). Thymus had the highest number of tissue-specific ASE genes (n = 15) followed by liver (n = 12), spleen (n = 5), and ileum (n = 4) (**Figures 4B–E**). Among the thymus gene set was *CD244*, which included 30 heterozygote loci with allelic imbalance, one of which was rs406633825. This missense allele (Chr1:110308273 C > A; pVal123Phe MAF = 0.3, SIFT score = 0 deleterious) has previously been reported in the Texel population characterized by the International Sheep Genome Consortium (ISGC) (Kijas et al., 2012). The CD244 protein molecule, a non-MHC (major histocompatibility complex)-mediated marker expressed by NK cells and multiple subsets of CD8+ T cells is known for both pro-inflammatory and inhibitory effects on lymphocytes (McNerney et al., 2005; Georgoudaki et al., 2015). *CD244* exons 2 to 5 are highly conserved in vertebrates and in mouse a trypanosome infection model indicated differential expression was correlated with multiple-copy number variants nearby (Goodhead et al., 2010).The liver-specific ASE profile included genes involved in amino acid metabolism, cytochrome oxidase pathways and fibrinogen: *FGA* (fibrinogen alpha chain), *ENSOARG00000003175* (taurochenodeoxycholic 6 alpha-hydroxylase-like), *ENSOARG00000001568* (novel gene, complement C4-A-like), *CYP3A24* (cytochrome P450 CYP3A24), and *CA3* (carbonic anhydrase 3). Allelic imbalance in spleen was present in *CACYBP* (calcyclin binding protein), *DAPK2* (death-associated protein kinase 2), and a novel gene *GIMAP8*-like (ENSOARG00000001131). The ASE in GIMAP8 has been previously reported in cattle with a strong paternal parent-of-origin expression pattern (Chamberlain et al., 2015). The proteins derived from the *GIMAP/IAN* gene family, are involved in survival, selection, and homeostasis of lymphocytes (Nitta and Takahama, 2007).

Two genes of functional interest showed evidence of strong tissue-specific ASE in the spleen: *SNAP23* and *MYLK*. SNAP23 protein is a key molecule in vesicle transport machinery of the cell and has been reported to be expressed in sheep spleen. SNAP23 or Synaptosome-Associated Protein 23 is part of the protein complex involved in class 1 MHC-mediated antigen processing and presentation and in neutrophil degranulation (Fabregat et al., 2018). *SNAP23* gene is also vital to lymphocyte development (both B and T) *in vitro* (Wong et al., 1997; Kaul et al., 2015). The myosin light chain kinase (*MYLK*) expression in the splenic trabeculae’s smooth muscle has been demonstrated previously (Jiang et al., 2014; Clark et al., 2017). Overall, 199 heterozygote bi-allelic loci were present within the *MYLK* gene. The variant rs400678033 (Chr1:186347056G > A;.pAla1014Val), a missense SNV in exon 17 of 33 exons in *MYLK*, showed consistent allelic imbalance in all spleen samples.

In summary, analysis of ASE across immune-related tissues revealed there were a small number of ASE genes that were shared across tissues. ASE signatures instead tended to be tissue-specific, within the sub-set of tissues investigated in this study.

### Individual-Specific ASE Signatures

**Figure 5:**
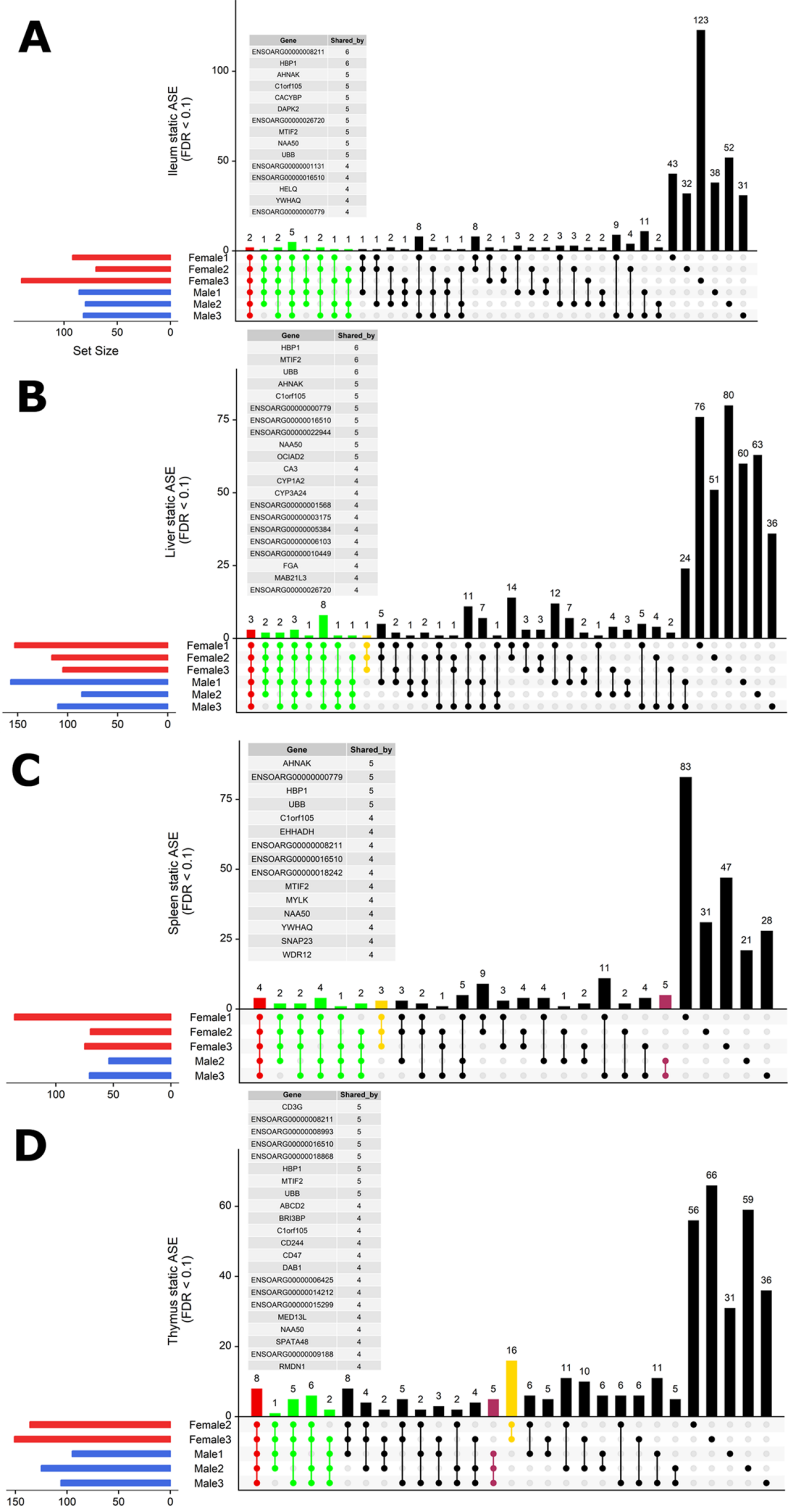
Intersectionality analysis of genes expressing significant ASE across all six sheep. In each tissue from left to right, the set count of genes (dots connected by lines) illustrates the number of sheep sharing the gene. The private sets of genes are located at the far right of each graph (single dots with no line). The intersections are colored in to illustrate the size of the set of shared genes (red [common to all six sheep], green [shared by five or four sheep], yellow [only in females] and purple [only in males]). Detailed lists of genes with ASE shared by at least four sheep are presented above each graph for **(A)** ileum, **(B)** liver, **(C)** spleen, and **(D)** thymus. Two sex-specific sets of genes are highlighted: 16 genes showing ASE only in females (in yellow) and five genes only in males (in purple).

To investigate whether ASE profiles were either shared across all six sheep or private to individual sheep, we used intersectionality (**Figure 5**). Each tissue was investigated separately. A number of private (to each individual) ASE genes were detected for each tissue, ranging from: 31–123 in ileum (**Figure 5A**), 24–80 in liver (**Figure 5B**), 21–83 in spleen (**Figure 5C**), and 31–66 in thymus (**Figure 5D**). Some of the shared sets of ASE genes in these tissues were specific to either male or female sheep, these sex-specific ASE signatures are described in **Figure 5**. In ileum, no sex-specific set was observed (**Figure 5A**). In contrast to ileum, the ASE profile for liver included a single gene with female only membership, *ENSOARG00000017409* (novel gene; 93% orthology with bovine dicarbonyl and l-xylulose reductase [*DCXR*]) (**Figure 5B**). In the spleen, all female sheep shared significant ASE in *PMS1* (PMS1 homolog 1, mismatch repair system component), *ANKRD10* (ankyrin repeat domain 10) and ENSOARG00000006103 which was not present in any of the spleen profiles of male sheep (**Figure 5C**). In the thymus, there were two sex-specific sets: 16 genes showing ASE only in females and five genes only in males. The female-specific thymus gene set included: *ARPP21* (cAMP regulated phosphoprotein 21), *CDKL3* (cyclin-dependent kinase-like 3), *CEP19 (centrosomal protein 19)*, *ENSOARG00000000710* (novel gene), *ENSOARG00000001163* (novel gene), *ENSOARG00000008981* (novel gene; t-lymphocyte surface antigen Ly-9-like), *ENSOARG00000006215*, *ENA000000008981*, *ENSOARG00000009129*, *ENSOARG00000011375* (blood vessel epicardial substance [*BVES*]), *ENSOARG00000015755*, *ENSOARG00000020354* (novel gene; 53% orthology with bovine monoacylglycerol acyltransferase [*MOGAT1*]), *ENSOARG00000025005*, *ENSOARG00000026030* (novel gene), *GPM6A* (glycoprotein M6A), *RAG1* (recombination activating 1), *STX8* (syntaxin 8). The male-specific thymus set was comprised of *ENSOARG00000007267* (novel gene; t-cell surface glycoprotein *CD1a*-like), *ENSOARG00000016841* (novel gene; 98% orthology with bovine ATP synthase membrane subunit G [*ATP5MG*]), *ENSOARG00000007603*, *SNX25* (sorting nexin 25) and *LDHA* (L-lactate dehydrogenase A chain) **Figure 5D**.

In summary, very few ASE genes were shared across all sheep, and the majority of ASE profiles were private to each sheep. Sex-specific ASE signatures were also detected, but due to the small sample size (n = 3) in both cases, these should be interpreted with caution.

### ASE in Stimulated and Unstimulated BMDMs (0 h vs 7 h +LPS)

We examined inducible ASE after 7 h of exposure to LPS in BMDMs using the ICD mode of GeneiASE (Edsgärd et al., 2016). A comparison of LPS-inducible ICD-ASE genes and the genes with background static ASE at 0 and 7 h timepoints, was also performed. We first assessed whether differences between 0 and 7 h could be observed using analysis of static ASE. Individual-specific ASE profiles and a limited number of shared ASE genes were also observed in BMDMs. The total number of genes with static ASE in the BMDMs is shown in **Table 2**.

**Table 2:** Total number of genes with significant static ASE in BMDMs ± LPS.

Sheep	BMDM 0 h −LPS	BMDM 7 h +LPS
Female 1	237	252
Female 2	192	233
Female 3	227	263
Female avg.	219	249
Male 1	205	193
Male 2	300	260
Male 3	260	261
Male avg.	255	238

Shared static ASE across both timepoints and independent of LPS induction was only observed in five genes. These genes have a macrophage associated function and include *ITGB2* (Yee and Hamerman, 2013), *SAA3* (*ENSOARG00000009963*) (Larson et al., 2003; Deguchi et al., 2013), *CD200R1* (*ENSOARG00000019357*) (Ocaña-Guzman et al., 2018), *DCTN5* (*ENSOARG00000017281*) (Habermann et al., 2001), and *MTIF2* (also seen in the tissue analysis above) (Overman et al., 2003).

**Figure 6:**
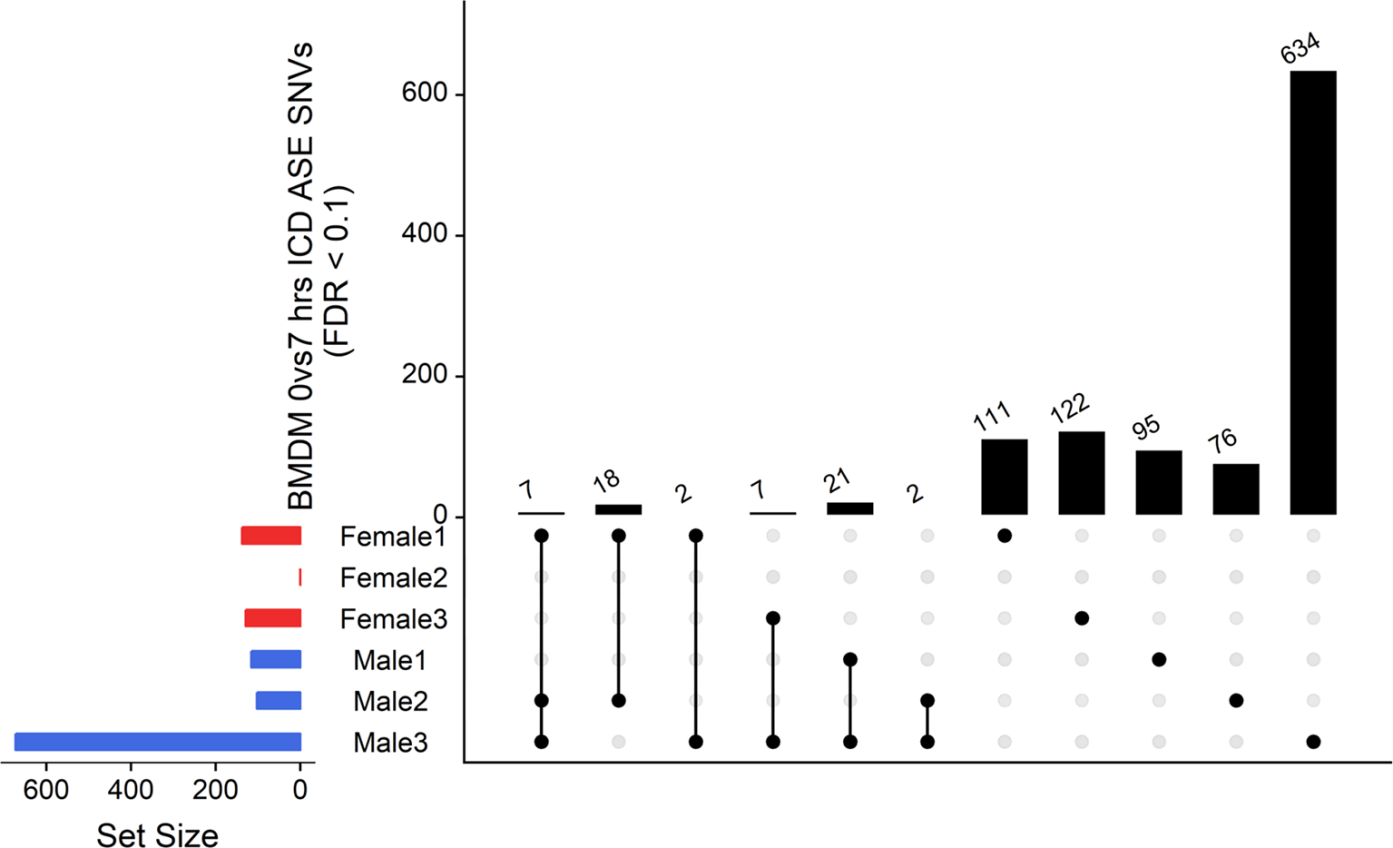
Intersection analysis of SNVs under genes with significant ICD-ASE in the BMDMs ± LPS. From left to right, the set number of genes (dots connected by lines) has been illustrated in order according to the number of sheep sharing the SNV. The private sets of SNVs are located at the far right of each graph (single dots with no line).

The ICD-ASE in BMDMs ± LPS captured fewer ASE genes with significant LPS-inducible ASE between the two timepoints than the static analysis of ASE. Moreover, there were large differences in the number of LPS-inducible ASE genes in each individual sheep, indicating significant individual-specific variation in response to LPS stimulation. BMDM cultured from female 2 showed no LPS-inducible response in comparison to male 3 which was a hyper responder with significant inducible ICD-ASE in 28 genes (including 634 informative SNVs total). A detailed breakdown of SNVs, aggregated within each gene, with significant ICD ASE has been summarized in **Figure 6**.

In summary, the ICD-ASE mode of GeneiASE’s model was not capable of capturing a complete picture of differential ASE in the BMDM experiment. Static ASE was present in both timepoints; however, there were no inducible ASE genes that were shared across all six sheep. The highly diverse ASE profile of BMDMs was very individual-specific, similar to patterns observed in tissues (**Supplementary Table S1**). These individual-specific differences could be due to individual variation in the innate immune response or experimental variation introduced during primary cell culture or stimulation with LPS.

### Condition-Dependent ASE at the SNV Level in BMDMs

**Figure 7:**
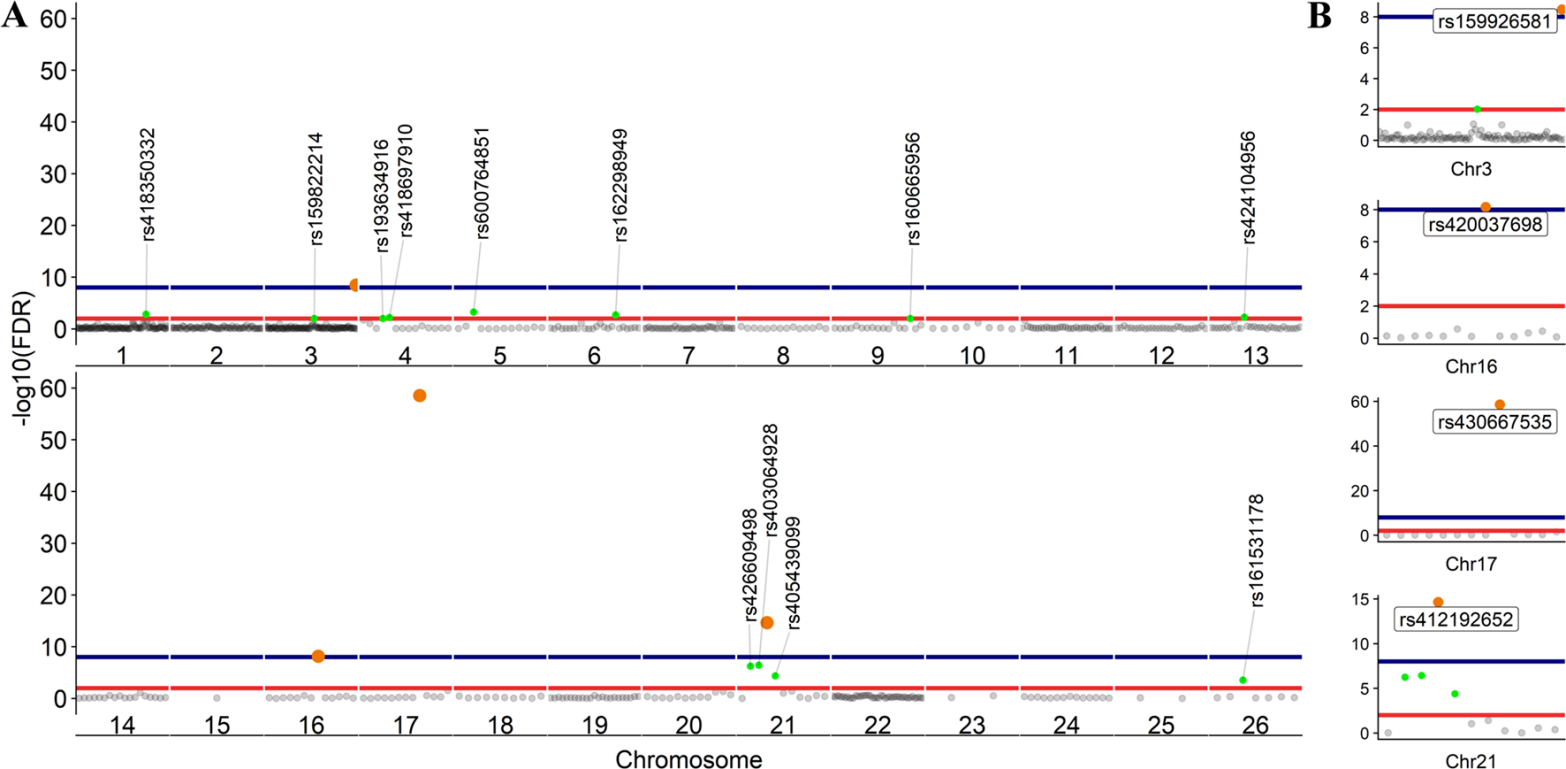
Scatter plot of the adjusted *p* values from Fisher’s exact test (unified using Stouffer unification) in BMDMs comparing expression from different alleles at 0 vs 7 h at SNV level (LPS-inducible ASE). **(A)** The graph shows 646 loci exhibiting LPS-inducible allelic imbalance shared across all six sheep. **(B)** Four loci on chromosomes 3, 16, 17, and 21 with false discovery rate (FDR) < 1 × 10^−8^. FDR < 1 × 10^−2^ red line (n = 16 SNVs) and FDR < 1 × 10^−8^ blue line (n = 4 SNVs).

To further investigate allelic imbalance at the SNV level without the aggregative gene model of GeneiASE, Fisher’s exact test was used. The filtered read counts for bi-allelic SNVs shared by all six sheep BMDMs were selected (n = 646 sites). Allelic read counts of each SNV were tested using Fisher’s exact test between 0 h and 7 h (2 × 2 table). Overall, the six sheep shared 646 SNVs with identical allelic genotypes in both timepoints of the BMDM RNA-Seq data set. These SNVs were tested for association with LPS treatment and only four SNVs showed a strong association (FDR < 1 × 10^−8^) and 12 SNVs had an FDR between 1 × 10^−2^ and 1 × 10^−8^ (**Figure 7**). The highest F-statistic was at rs430667535 Chr17 Pos:50485358T > C, a synonymous variant in ubiquitin C (*UBC*), a polyubiquitin precursor, and also an intronic variant T > C or A > G in pro-apoptotic *BRI3* binding protein (*BRI3BP*). This variant was shown to have a minor allele frequency (MAF) of 0.25 in Texel sheep based on the ISCG annotation [ISGC – Ensembl v.92] (Kijas et al., 2012). The next highest peak was observed on Chr21 under SNVs within *SAA3* gene boundaries (*ENSOARG00000009963*) at the following coordinates: highest FDR peak was observed at SNV rs412192652 (Pos:25826978A > G missense variant [Asn145Asp], FDR = 2.3 × 10^−15^) surrounded by rs403064928 (Pos:25826884C > A missense variant [Asp113Glu] in exon 4 of *SAA3* FDR = 3.6 × 10^−7^), rs426609498 (Pos:25826845A > G synonymous, FDR = 5.5 × 10^−7^) and rs405439099 (Pos:25826990G > C 3’UTR variant, MAF 0.4 in Texel sheep, FDR = 4.1 × 10^−5^). This region contains a strong LD block previously reported by the ISGC COMPOSITE population (Ensembl v.92) (**Supplementary Figure S4**), e.g., rs412192652 and rs405439099 pairwise D’ statistics = 1. Two further peaks on Chr3 were observed for rs159926581 (Pos:214731375T > C synonymous, FDR = 3.3 × 10^−9^) in ribosomal protein L3 (*ENSOARG00000016495*) and rs159822214 (Pos:112164732G > A 3’UTR variant, FDR = 9.5 × 10^−3^) in oxysterol binding protein like 8 (*OSBPL8*). The last inducible ASE associated signal was on Chr16 rs420037698 (Pos:6887423G > A, FDR = 6.9 × 10^−9^) in *ENSOARG00000004700*, a known synonymous variant in the Texel population (MAF = 0.55). The SNVs and corresponding genes from Fisher’s exact test are summarized in **Table 3**.

**Table 3:** The variant IDs of LPS-inducible ASE SNVs (Fisher’s exact) and their respective genes. Data were obtained using Ensembl BioMart query builder. Highly significant SNVs are highlighted in bold [false discovery rate (FDR), < 1×10^-8^].

ID	Chr	Position	Gene ID	Gene Name
rs418350332	1	195297663	ENSOARG00000020472	CLDN1
rs159822214	3	112164732	ENSOARG00000014876	OSBPL8
**rs159926581**	**3**	214731375	**ENSOARG00000016495**	–
**rs159926581**	**3**	214731375	**ENSOARG00000022372**	RF00221
**rs159926581**	**3**	214731375	**ENSOARG00000024737**	RF00593
**rs159926581**	**3**	214731375	**ENSOARG00000025124**	RF01151
**rs159926581**	**3**	214731375	**ENSOARG00000025150**	RF00593
rs193634916	4	53430934	ENSOARG00000001754	MDFIC
rs418697910	4	53430968	ENSOARG00000001754	MDFIC
rs162298949	6	92979793	ENSOARG00000018710	ANXA3
rs162298949	6	92979793	ENSOARG00000018710	ANXA3
rs160665956	9	76759747	ENSOARG00000001261	SPAG1
rs424104956	13	46654781	ENSOARG00000017292	CDS2
rs424104956	13	46654781	ENSOARG00000017292	CDS2
rs424104956	13	46654781	ENSOARG00000022618	RF00377
**rs420037698**	**16**	**6887423**	**ENSOARG00000004700**	–
**rs430667535**	**17**	**50485358**	**ENSOARG00000017707**	**BRI3BP**
**rs430667535**	**17**	**50485358**	**ENSOARG00000018177**	**UBC**
rs403064928	21	25826884	ENSOARG00000009963	SAA3
rs405439099	21	25826990	ENSOARG00000009963	SAA3
**rs412192652**	**21**	**25826978**	**ENSOARG00000009963**	**SAA3**
rs161531178	26	19663270	ENSOARG00000009626	MSR1

The LPS-inducible ASE analysis, of SNVs, using Fisher’s exact test revealed a different picture not captured by the gene level analysis with the GeneiASE model that aggregates SNVs within each gene (**Figure 6** vs **Figure 7**). The aggregative gene model did not capture any shared ASE genes in the ICD-ASE mode. Although the six sheep shared 646 SNVs and showed highly significant association with stimulation with the LPS (Fisher’s exact method), the aggregation of ASE effect size from SNV to gene level (ICD-ASE mode) only detected individual-specific sets of ASE genes in each sheep. This contradicted the results from Fisher’s exact test which detected four highly significant LPS-inducible shared regions (FDR < 0.01, 16 SNVs) on chromosomes 3, 16, 17, and 21. For example, the allelic imbalance in the *SAA3* genomic coordinates on chromosome 21 was not detectable in the ICD-ASE model but it was captured by the Fisher’s exact test in all individuals (**Figure 7B** chromosome 21).

Fisher’s exact analysis at SNV level revealed ASE in response to LPS in variants within *CLDN1*, *ANXA3*, *BRI3BP*, *SAA3*, and *MSR1* (**Table 3**). The anti-inflammatory macrophage marker *CLDN1* (Van den Bossche et al., 2012) and acute-phase inflammation resolution marker *ANXA3* (Yamanegi et al., 2018) have been previously reported with distinct macrophage functions. The macrophage scavenger receptor 1 (*MSR1*) has also been shown to be involved in lipid uptake and migration ability of macrophages (Shi et al., 2019). Three noncoding RNAs (*RF00221* [*snoRD43*], *RF00593* [*snoU83B*], and *RF01151* [*snoU82P*] were among genes corresponding to ASE inducible SNVs. These three snoRNAs all overlap with the genomic coordinates of ribosomal protein L3 (*ENSOARG00000016495/RPL3*). The *RF00377* [*snoU6-53*] was also among the ASE-positive targets which overlaps the protein-coding gene *CDS2* (CDP-diacylglycerol synthase 2). Using total RNA-Seq (ribosomal RNA depleted), which includes multiple RNA populations, to generate short read illumina data makes it difficult to pinpoint the origin of the ASE signal to a specific RNA population.

In summary, ASE profiles in BMDMs were highly individual-specific at both gene and SNV level. Moreover, Fisher’s exact SNV level analysis discovered shared ASE SNVs where the aggregative gene model of ICD-ASE mode did not, indicating for condition-dependent ASE analysis Fisher’s exact test is more accurate and robust at SNV level.

## Discussion

This study is the first to investigate global allele-specific expression across tissues from sheep using RNA-Seq data. We focused our analysis on immune-related tissues and cell types from six adult crossbred sheep (T×BF) from the sheep gene expression atlas. ASE profiles were highly individual-specific in the six sheep analyzed in this study. We were able to identify tissue-specific sets of ASE genes, as well as LPS-inducible sets in the BMDM experiment. Tissue-specific signatures of ASE have been previously reported in similar studies in mouse (Castel et al., 2015; Castel et al., 2016), goat (Cao et al., 2019) and cattle (Chamberlain et al., 2015).

Several steps were taken in the cattle study (Chamberlain et al., 2015) to mitigate the ref allele bias, assign parental origin using whole genome sequences and include MAE variants. The SNV filtration was based on the (Hayes and Daetwyler, 2019) 1000 bull genomes project to confirm the heterozygote sites. In our pipeline, the Ensembl VCF track was used for that purpose. Chamberlain et al. (2015) use a 0.9 allele frequency cutoff (based on read counts) to define and include MAE, and as such have a 1 and 0 inflated allelic ratios. In our pipeline, no allelic ratio cutoff is introduced for inclusion as it is difficult to distinguish between sequencing error and MAE. The minimum read (bi-allelic expression) filtration criteria was applied to exclude highly sequenced loci (Either count/Total >1%) or sequencing errors presenting as rare alleles (min either allele count ≥ 3) which consequentially excludes actual MAE as well as spurious allelic counts. Chamberlain et al. tested 5317 genes (14,495 SNVs) in spleen and detected 382 ASE genes (with min > 1 SNV per gene, similar to this study). Although direct comparison would not be appropriate (because we have excluded MAE variants in our analysis), our analysis of sheep spleen revealed ASE in 86 genes (averaged in five sheep) from 8272 filtered genes (averaged in five sheep). Similarly, in the thymus, the cattle study showed 182 ASE genes from 986 informative genes (9781 SNVs), whereas from 7961 filtered genes in sheep thymus, 134 ASE genes were captured. The differences in the numbers of genes exhibiting ASE between the two studies are likely to be a consequence of the filtration criteria applied, the exclusion of MAE, and species-specific differences between sheep and cow. Results from a more recent study in goat (Cao et al., 2019) more closely reflect our findings. They apply similar filtration criteria to our workflow and discovered 144 ASE genes in liver in comparison to 123 in our sheep liver sets (averaged across six sheep). Other recent studies, including those focusing on production relevant tissues, such as muscle (Guillocheau et al., 2019), have also applied similar stringency in filtration criteria. The filtering criteria we have used for this analysis is stringent and focused on detecting variants of moderate to extreme effects. Further analysis of the data set reducing these criteria might discover additional variants exhibiting ASE across individuals and tissues, but it would also increase the potential risk of false-positive discovery.

For this analysis, we have adapted an ASE analysis workflow with a primary focus on mapping bias removal prior to allele-specific analysis of the transcriptome. The collection of scripts for WASP, used for this analysis, or modified versions of them have been utilized by others for mapping bias removal in reference-guided genomic data sets, e.g., RNA-Seq (Mozaffari et al., 2018; Zhou et al., 2018), Chip-Seq (Pelikan et al., 2018), and for methylomic and epigenetic analysis (Richard Albert et al., 2018).

The ASE analysis pipeline we have adapted for sheep for this study is also adaptable to other species and tissue types with available RNA-Seq data sets. It could be applied, for example, to profile allele-specific expression in the RNA-Seq data sets from livestock species listed on the FAANG data portal (Andersson et al., 2015; Harrison et al., 2018). We used the Ensembl VCF track to capture information at heterozygote loci; however, the individual VCF file from each sheep could also be used in ASE analysis. The latter strategy might enable the capture of rare variants not included in the publicly available VCF tracks but would also raise the issue of normalization/standardization between VCF call sets. The usage of either of these methods will be limited to the number of loci shared by coordinate and bi-allelic genotype (i.e., pervasive ASE discovery). Other studies have compared variants at the RNA and DNA level from the same individual and then removed the DNA variants not present in the RNA-Seq data from the analysis (Guillocheau et al., 2019). We believe that the strength of the pipeline we present is that it does not require parental genotypes and can therefore be applied to other RNA-Seq data sets for livestock where this information is not available.

In our analysis we have not considered either parent-of-origin or breed-of-origin-specific effects in this analysis. For parent-of-origin or breed-of-origin assignment of these ASE profiles, DNA level genotypes from the parents of the six sheep from the gene expression atlas (i.e., Texel sire and Scottish Blackface dam) would be required, and these are unfortunately not available. In this study, ASE expression profiles also might be affected by the direction of the cross (i.e., Texel sire × Scottish Blackface dam). To fully characterize parent-of-origin or breed-of-origin, reciprocal cross experiments would be required. Reciprocal cross studies in mouse (Huang et al., 2017), chicken (Zhuo et al., 2017), and crossbred cattle (Chen et al., 2016b) have shed light on the complexity of such pervasive ASE markers and parent-of-origin effects. Though potentially very interesting, these experiments are lengthy and costly to perform in sheep. Particularly, in this case, because the reciprocal cross (Scottish Blackface dam × Texel sire) is rarely used in the UK sheep industry and as a consequence has limited relevance to production.

Our approach also excludes mono-allelic expression. The minimum filtration criteria utilized in our workflow along with the reference mapping bias removal step ensures an unbiased ASE discovery in the transcriptome by excluding the ambiguity surrounding MAE variants. This form of analysis is based on the principle that absence of evidence (reads) for either allele of a heterozygote site does not directly amount to evidence of their absence, i.e., MAE. The pattern of ASE (ratio of Alt/Ref+Alt) is dependent on the bi-allelic expression of loci within the genomic coordinates of the gene or genomic element of interest. For an ASE effect to be captured by the GeneiASE model, the following criteria must be met: (i) biallelic expression of the locus; (ii) min depth criteria for each allele (min 3 reads, total 10 reads at that site and > 1% of total reads containing that allele); (iii) the allelic imbalance or departure from bi-allelic balanced expression being inducible by an environmental trigger (i.e., LPS in ICD ASE experiment with BMDM data). These stringent criteria secure robust transcriptome-wide ASE discovery while maximizing the usage of read counts from short read RNA-Seq data sets without considering mono-allelic sites. MAE patterns are impossible to differentiate from sequencing error or random nonsense-mediated decay in total RNA-Seq, unless arbitrary cutoffs are introduced, such as ratio of allelic read counts > 0.9 (Chamberlain et al., 2015) or > 0.7 (Cao et al., 2019). We decided to exclude MAE from this study using read count bi-allelic expression filtration because it is difficult to distinguish between sequencing error and MAE. We do appreciate that this form of filtration might lead to a reduced number of ASE genes discoveries overall and will exclude potentially imprinted loci altogether.

The tissues utilized for ASE analysis in this study (thymus, spleen, liver, and ileum) are highly influential on performance of the immune system. ASE profiles shared across tissues and cell types were limited and instead they tended to be highly specific. We identified tissue-specific ASE in several genes in the thymus, for example, those that are involved in the T cell–mediated immune response, including *CD47* and *CD244*. These tissue-specific and cell-type–specific ASE profiles may underlie the expression of economically important traits, such as disease resistance. Assessment of the connection between economically relevant phenotypes and tissue-specific ASE profiles could be useful for the improvement of genomics enabled sheep breeding programs, particularly those using specialized sire and dam lines (Georges et al., 2018). Loci exhibiting ASE have been associated with production traits including milk-fat percentage (Hayes et al., 2010; Suárez-Vega et al., 2017), trypanotolerance in small ruminants (Kadarmideen et al., 2011; Álvarez et al., 2016), mastitis in goat (Ilie et al., 2018), Johne’s disease in cattle (Mallikarjunappa et al., 2018), and Marek’s disease in chicken (Maceachern et al., 2011; Meydan et al., 2011; Cheng et al., 2015). Although there is no general consensus currently on the correlation of allelic expression haplotypes and phenotypes under selection in sheep, this form of ASE analysis could pave the way for functional validation at population level (e.g., breed or haplotype-specific aseQTL studies in a larger population of sheep). Examples of population level aseQTL, eQTL, and sQTL (QTLs associated with RNA splicing) already exist for cattle (Wang et al., 2018; Xiang et al., 2018). Knowledge of favorable ASE in critical genes involved in traits of interest could be used as a performance indicator or included as weighted SNVs in genomic prediction algorithms to enhance livestock breeding programs (Georges et al., 2018). Currently, the UK sheep industry is on the cusp of applying genomic prediction, but suitable genomics enabled breeding programs for sheep already exist in New Zealand and Australia (Daetwyler et al., 2010).

## Conclusions

In this study, we characterize extreme to moderate allele-specific expression, at the gene and SNV level, in immune-related tissues and cells from six adult sheep (T×BF) from the sheep gene expression atlas data set. Reference mapping bias removal was an integral component of the analysis pipeline applied in this study. The correction of reference bias prior to obtaining the allelic read counts is a critical step toward true ASE discovery. The workflow developed as part of this manuscript provides an RNA-Seq-only–dependent tool, without the need for individual DNA sequences. We note that the stringent filtering process applied would remove loci where the allelic imbalance was less extreme but might still be of biological significance.

This study is a novel analysis of an existing large-scale complex RNA-Seq data set from sheep. Using the pipeline, we have adapted for this analysis, we were able to identify ASE profiles that were pervasive in each sheep and specific to the tissues and cell types investigated. These tissue and cell type-specific ASE profiles may underlie the expression of economically important traits and could be used to identify variants that could be weighted in genomic prediction algorithms for the improvement of sheep breeding programs. In summary, we have adapted a robust methodology for ASE profiling, using the sheep gene expression atlas data set, and provided a foundation for identifying the regulatory and expressed elements of the genome that are driving complex traits in livestock.

## Data Availability

The RNA-Seq sequence data are available *via* the European Nucleotide Archive (ENA) under PRJEB19199 (https://www.ebi.ac.uk/ena/data/view/PRJEB19199). The BAM files were already produced as part of the sheep gene expression atlas following the publicly available protocol (FAANG, 2018) and as described in (Clark et al., 2017). The BAM files have been uploaded to ENA under accession numbers ERZ827944, ERZ827949, ERZ827951, ERZ827955, ERZ827972, ERZ827988, ERZ827995, ERZ827997, ERZ828001, ERZ828016, ERZ828019, ERZ828036, ERZ828044, ERZ828046, ERZ828050, ERZ828070, ERZ828073, ERZ828160, ERZ828167, ERZ828168, ERZ828172, ERZ828188, ERZ828192, ERZ828209, ERZ828215, ERZ828217, ERZ828221, ERZ828240, ERZ828244, ERZ828261, ERZ828268, ERZ828270, ERZ828274, ERZ828293, and ERZ828297. The Oar v3.1 reference FASTA and VCF file from Ensembl v92 were used throughout the pipeline. The ASE analysis pipeline (https://msalavat@bitbucket.org/msalavat/asewrap_public.git) was wrapped using bash scripting on Edinburgh Compute and Data Facility computing resource Eddie Mark 3 (Edinburgh, 2018). All the raw ASE genes data produced by GeneiASE are included in **Supplementary File 2** (Supplementary_file2.zip). The comparison of ASE positive SNVs between imbalance towards the ref or alt alleles is detailed in **Supplementary Figure S7**. The clustering behavior of ASE profiles in BMDMs experiment were explored using Kmeans clustering and Principle Component Analysis (PCA) as shown in **Supplementary Figure S8** and **S9** respectively. All the supplementary material are available at (https://doi.org/10.6084/m9.figshare.8035799.v1).

## Ethics Statement

Approval was obtained from The Roslin Institute, University of Edinburgh’s Animal Work and Ethics Review Body (AWERB). All animal work was carried out under the regulations of the Animals (Scientific Procedures) Act 1986.

## Author Contributions

MS and EC coordinated and designed the analysis component of the study with assistance from SB and SP-V. MS and SP-V designed, optimized and tested the ASE pipeline. DH acquired the funding for the sheep gene expression atlas project. MM, EC, and DH designed the LPS experiment and generated the data. MM performed the LPS stimulation of bone marrow derived macrophages and RNA extraction. SB performed all bioinformatic analyses prior to analysis with the ASE pipeline. MS performed ASE analysis, visualization of the results and wrote the manuscript. EC contributed to manuscript editing and drafting. All authors read and approved the final manuscript.

## Funding

The work was supported by a Biotechnology and Biological Sciences Research Council (BBSRC) (http://www.bbsrc.ac.uk) Grant BB/L001209/1 (‘Functional Annotation of the Sheep Genome’). Also, BBSRC Institute Strategic Program Grants: ‘Farm Animal Genomics’ (BBS/E/D/20211550), ‘Transcriptomes, Networks and Systems’ (BBS/E/D/20211552), ‘Improving Animal Production and Welfare’ (BB/P013759/1) and ‘Blue Prints for Healthy Animals’ (BB/P013732/1). Edinburgh Genomics is partly supported through core grants from BBSRC (BB/J004243/1), NERC (http://www.nerc.ac.uk) (R8/H10/56) and the Medical Research Council (MRC) (https://www.mrc.ac.uk) (MR/K001744/1). SB was supported by the Roslin Foundation. EC is supported by the University of Edinburgh Chancellor’s Fellowship programme. The funders had no role in the study design, data collection and analysis, decision to publish, or preparation of the manuscript.

## Conflict of Interest Statement

The authors declare that the research was conducted in the absence of any commercial or financial relationships that could be construed as a potential conflict of interest.
