# Supplementary material for: Elimination of Reference Mapping Bias Reveals Robust Immune Related Allele-Specific Expression in Crossbred Sheep
Source: Front Genet. 2019 Sep 19;10:863. doi: 10.3389/fgene.2019.00863 (PMC6761296; doi:10.3389/fgene.2019.00863)
Supplement: Supplementary file 1 [file Supplementary_file1_new.pdf]

Supplementary material

A bimodal p value distribution was observed in GeneiASE output as shown in S1 Fig.1:

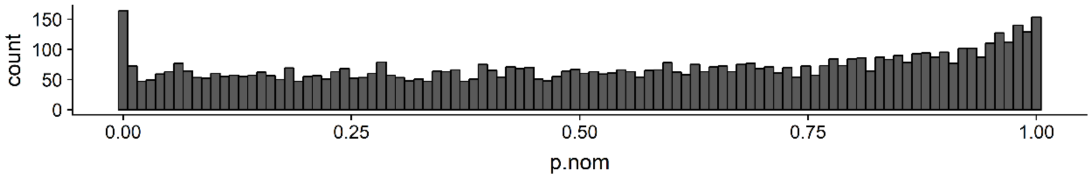

Figure S1. Distribution of p values resulted from the beta-binomial test (Static ASE example for spleen in female 1). The uniformity of the p value distribution showed a bimodal (high frequency close to 1 followed by an extensive lower tail close to 0). R machine characteristics for printing float numbers were limited to 6 significant numbers after the decimal point by default which resulted in a high number of absolute zero (0+e0.0) output-values.

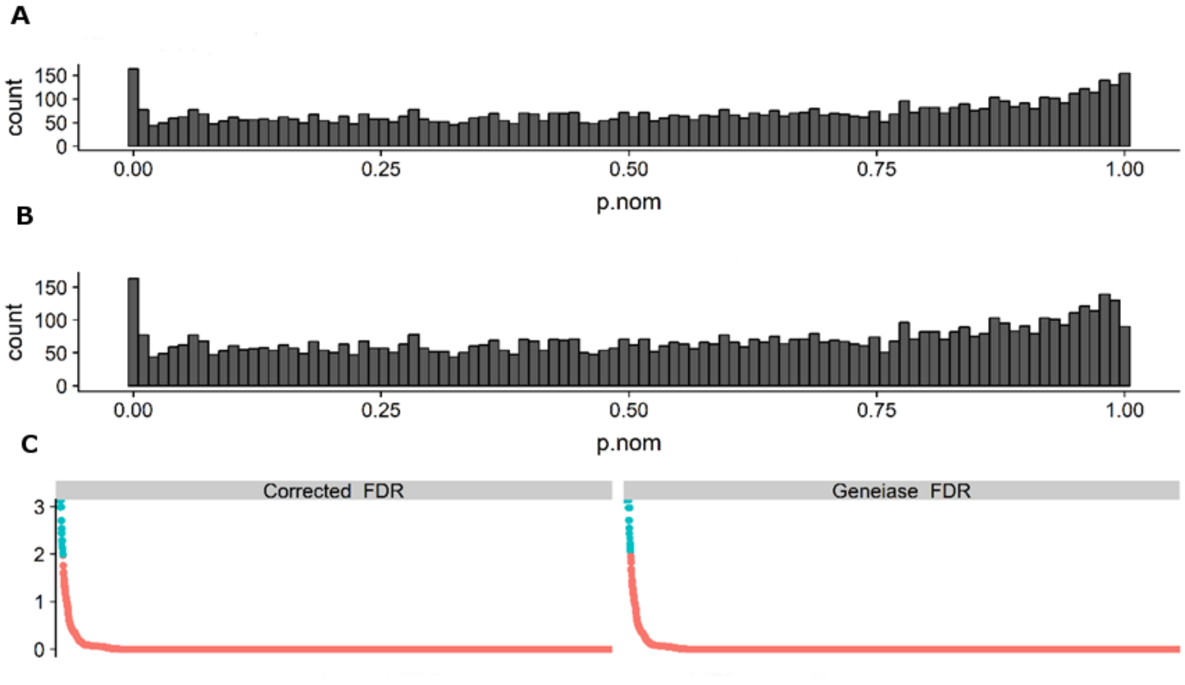

Figure S2. The figure shows the distribution of p values produced by GeneiASE after cut off introduction (A) and after removal of p value == 1 values (B). The distribution of sorted FDR calculated by GeneiASE and after correction are shown in C (right and left respectively). To correct for multiple testing by recalculation of FDR and eliminating the non-uniformity of p value distribution around 1 the following steps were performed as shown in S1 Fig.2: i) An arbitrary cut off (1e-7) was placed over all p values smaller than the said limit including the absolute 0e+0.0; ii) The genes showing p values equal to 1 were removed from each data frame; iii) The false discovery portion and rate were recalculated in each sample. iv) Gene showing FDR < 0.1 selected for further analysis.

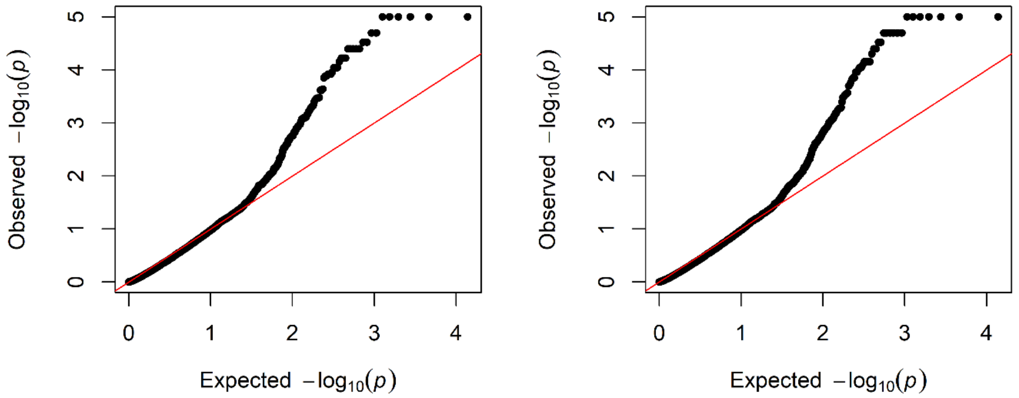

Figure S3. The QQ plot of observed vs expected p values in corrected (left) and GeneiASE's raw (right) calculated outputs.

A

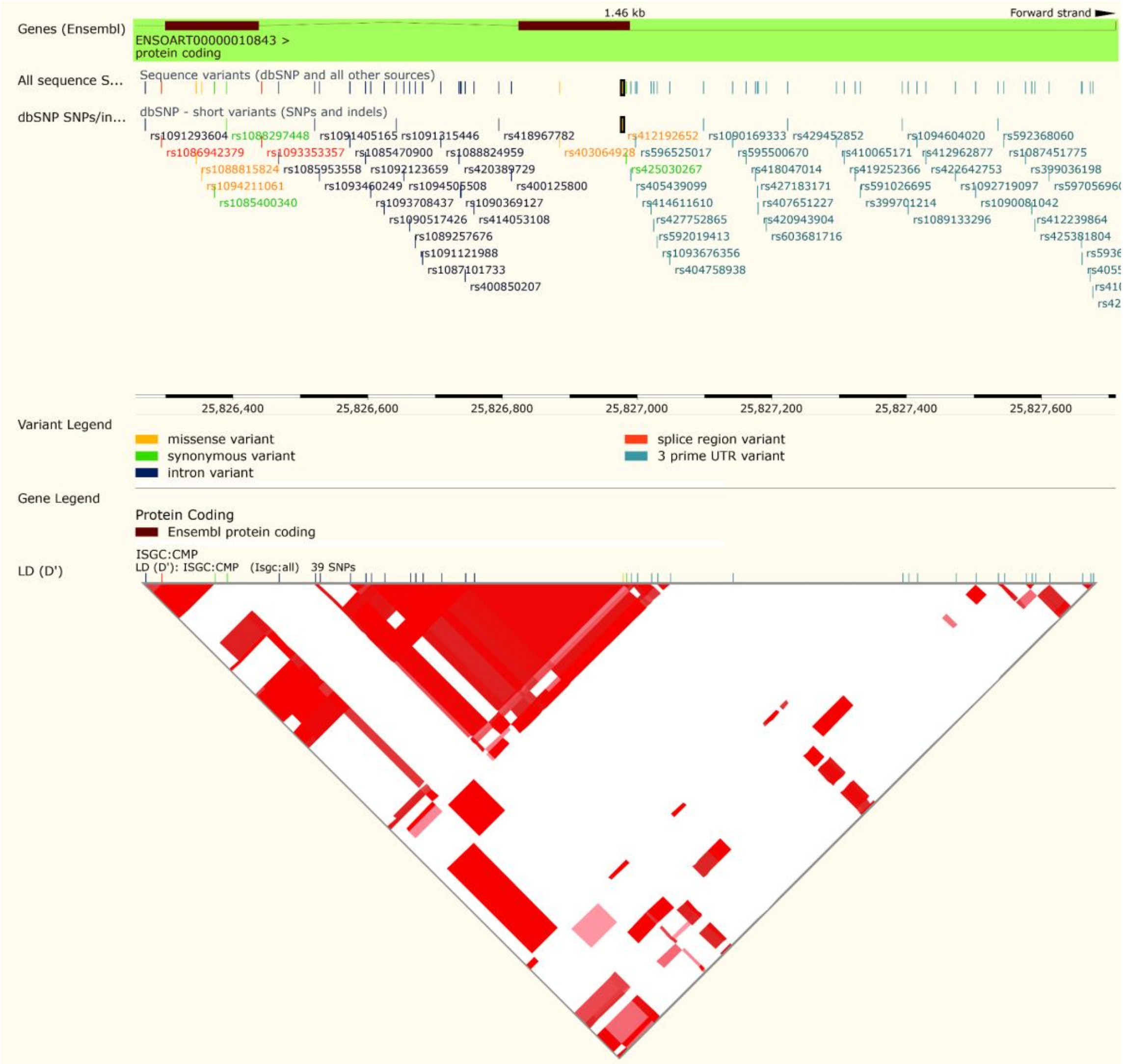

B

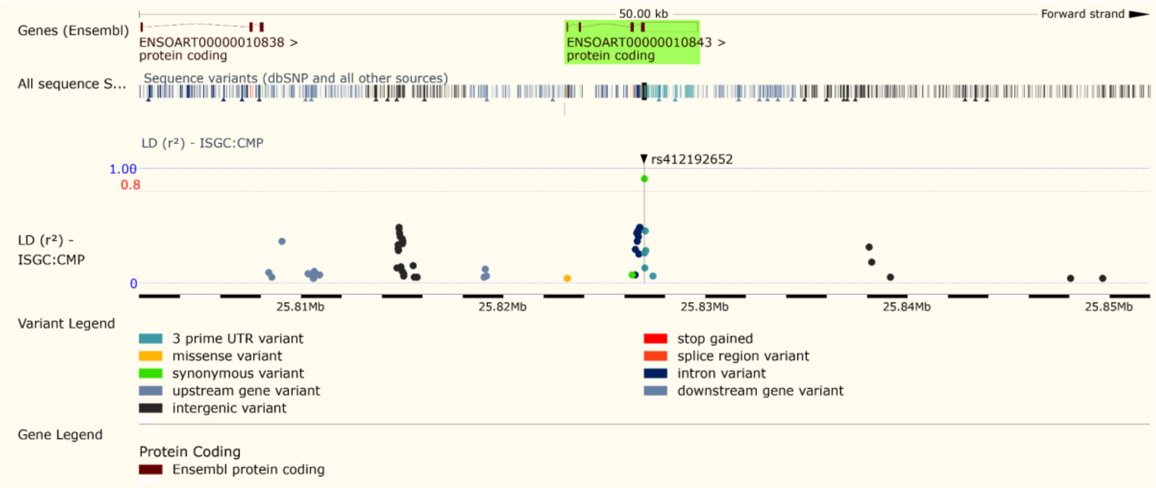

Figure S4. The LD block (A) and LD Manhattan plot (B) of SAA3 transcript (ENSOART00000010843). The LD block produced as part of International Sheep Genome Consortium is publicly available on Ensembl using the COMPOSITE population (n = 98).

A

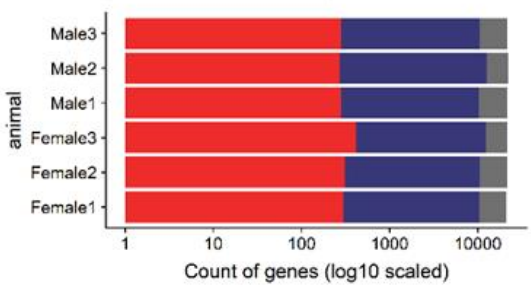

B

animal	Feature	Expressed	Filtered	ASE
Female1	gene	21150	10379	297
Female1	snv	6151417	125210	5985
Female2	gene	21420	10572	310
Female2	snv	5837076	109205	7095
Female3	gene	21689	12326	417
Female3	snv	5961473	137852	10811
Male1	gene	21361	10282	279
Male1	snv	5862189	125651	6740
Male2	gene	21822	12514	271
Male2	snv	6438497	151451	7110
Male3	gene	21545	10480	279
Male3	snv	5673703	115660	6558

Figure S5. The proportional distribution of ASE genes and SNVs being expressed across tissues averaged for each sheep. A) Count of genes in 6 sheep expressed in grey, informative/filtered in blue and ASE positive in red. B) Break down of genes and SNVs that are expressed, pass minimum filtration criteria and are ASE positive.

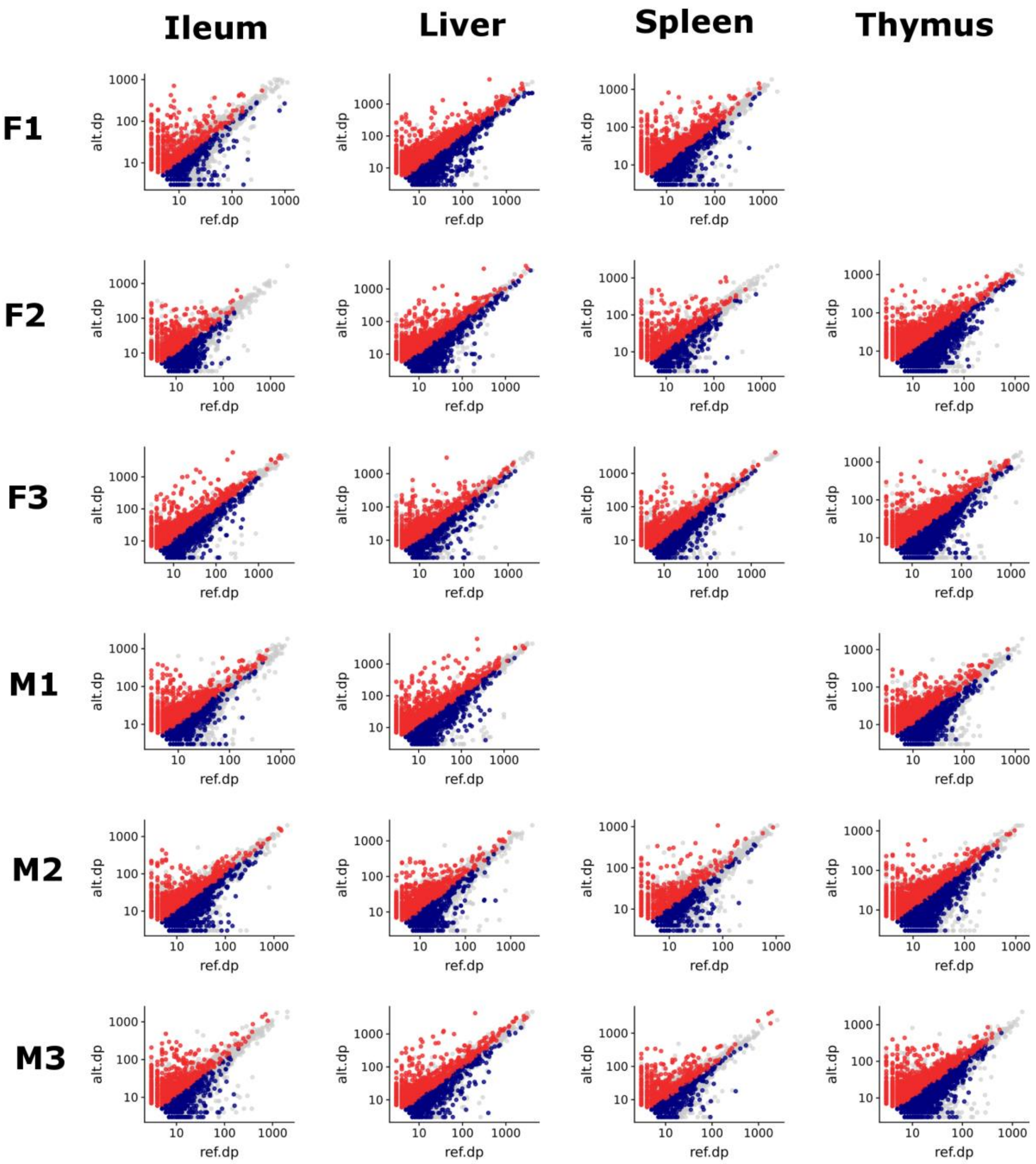

Figure S6. Scatter plot of allelic depth (read count) in all 4 tissues of the 6 TxBF sheep. The SNVs passing minimum filtration criteria are coloured in grey and significant ASE (FDR10%) are marked by red and blue. If reference read count > alternate read count the point is shown in blue and vice versa in red. Expression level varies across tissues but does not affect the ASE profile.

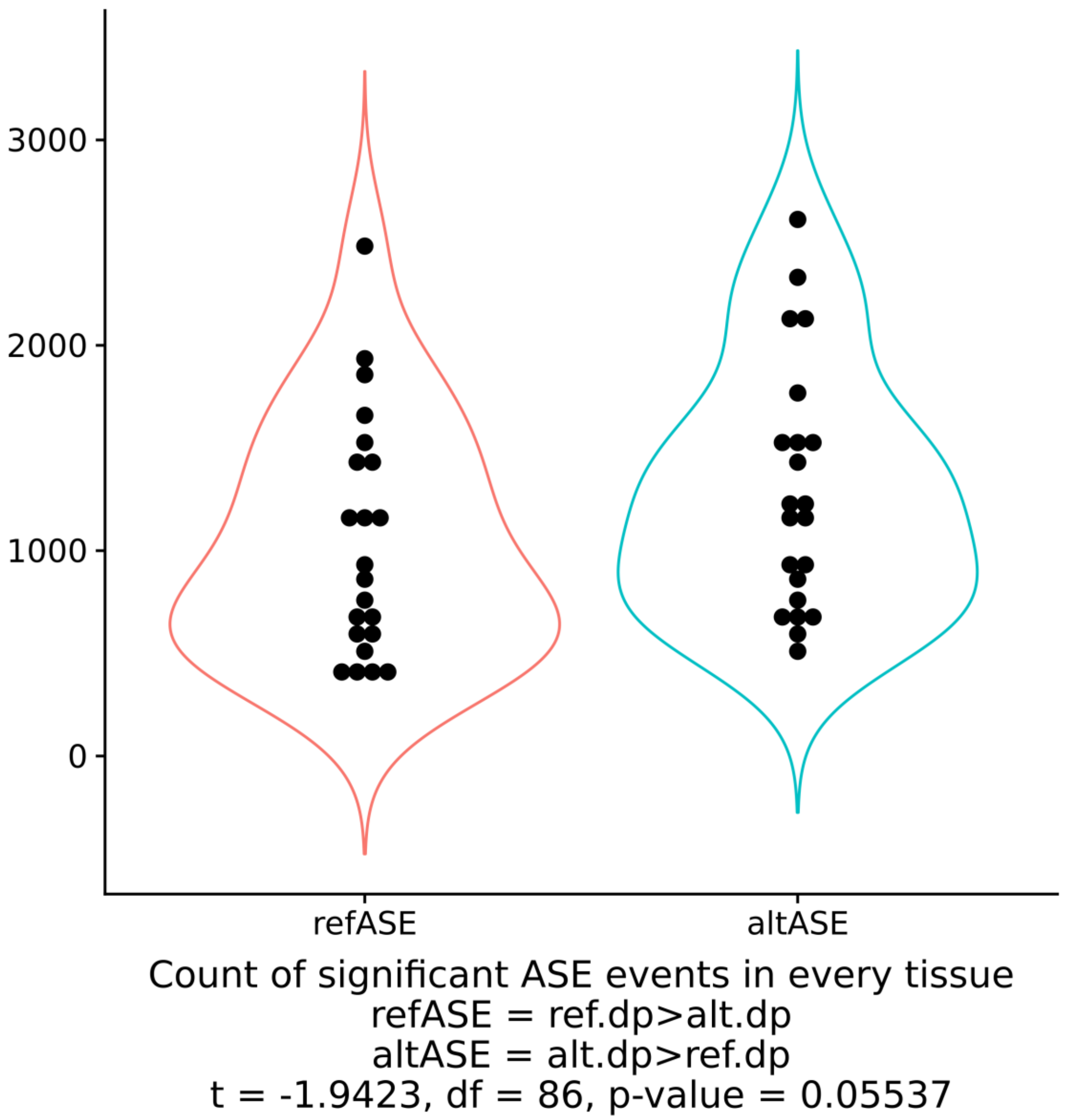

Figure S7. Distribution of ASE positive SNVs over the direction of imbalance in the 34 tissues of 6 sheep. Ref.dp > Alt.dp as ASE towards Ref allele and vice versa. There was no significant difference between the count of ASE events given the direction of the allelic imbalance (p-value > 0.05).

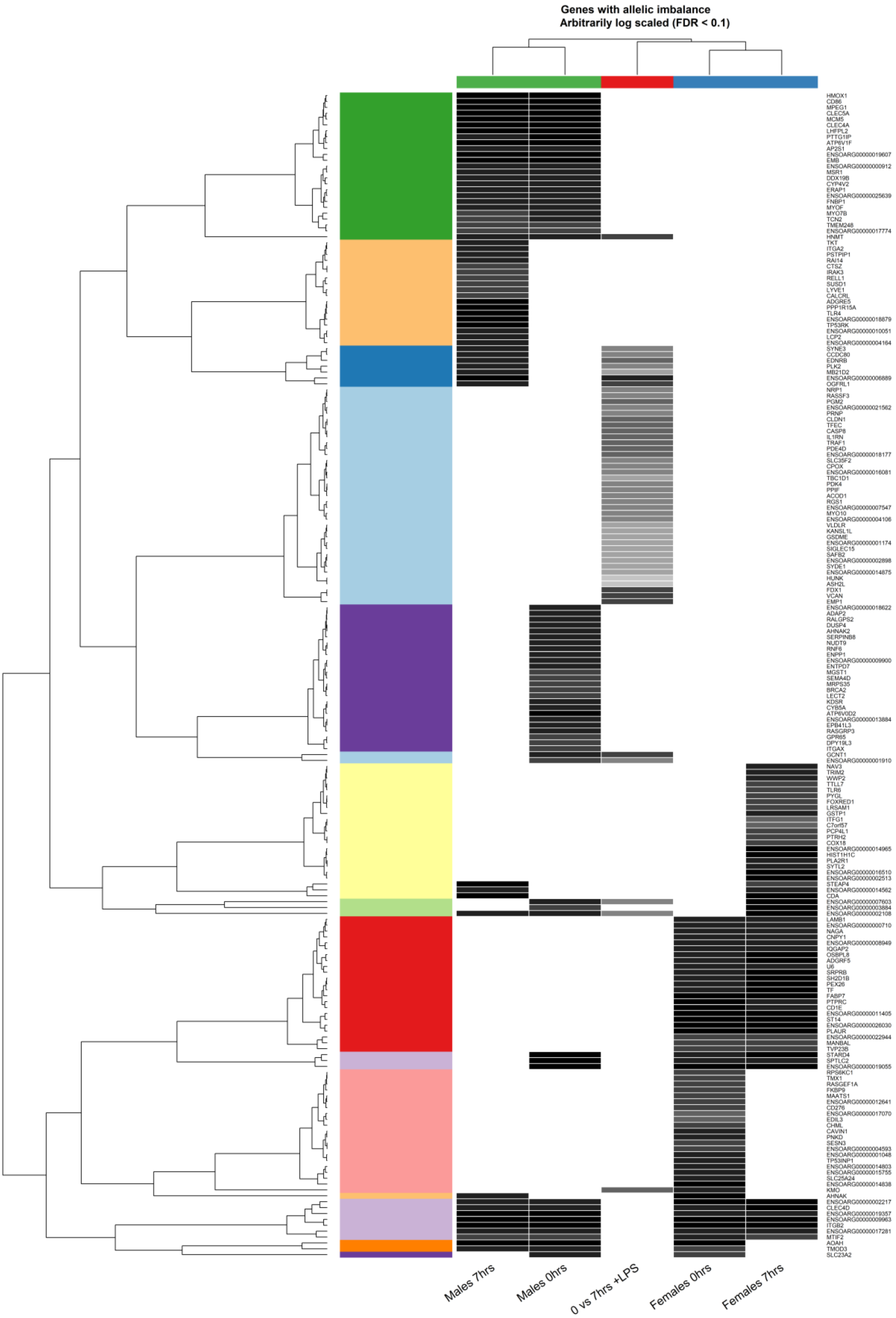

Figure S8. The Kmeans cluster analysis of the ASE genes in between static and ICD profiles of BMDMs at 0 and 7hrs samples. Heat map dataframe has been log and centre

scaled prior to cluster analysis. The heatmap shows the ASE genes in 5 groups: 0vs 7 hrs ICD set, Males 0hrs , Males 7hrs . Females 0hrs and Females 7hrs.

A

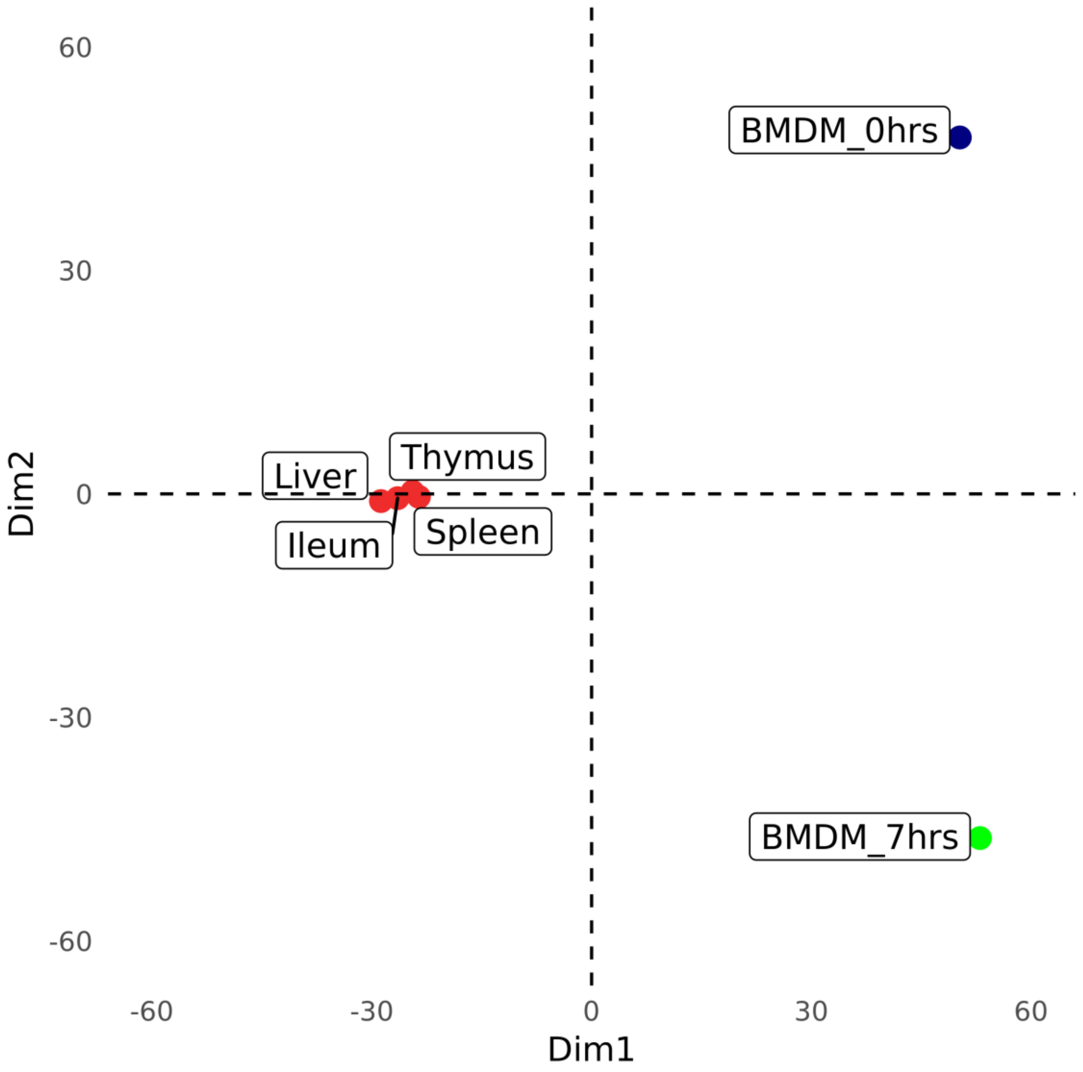

B

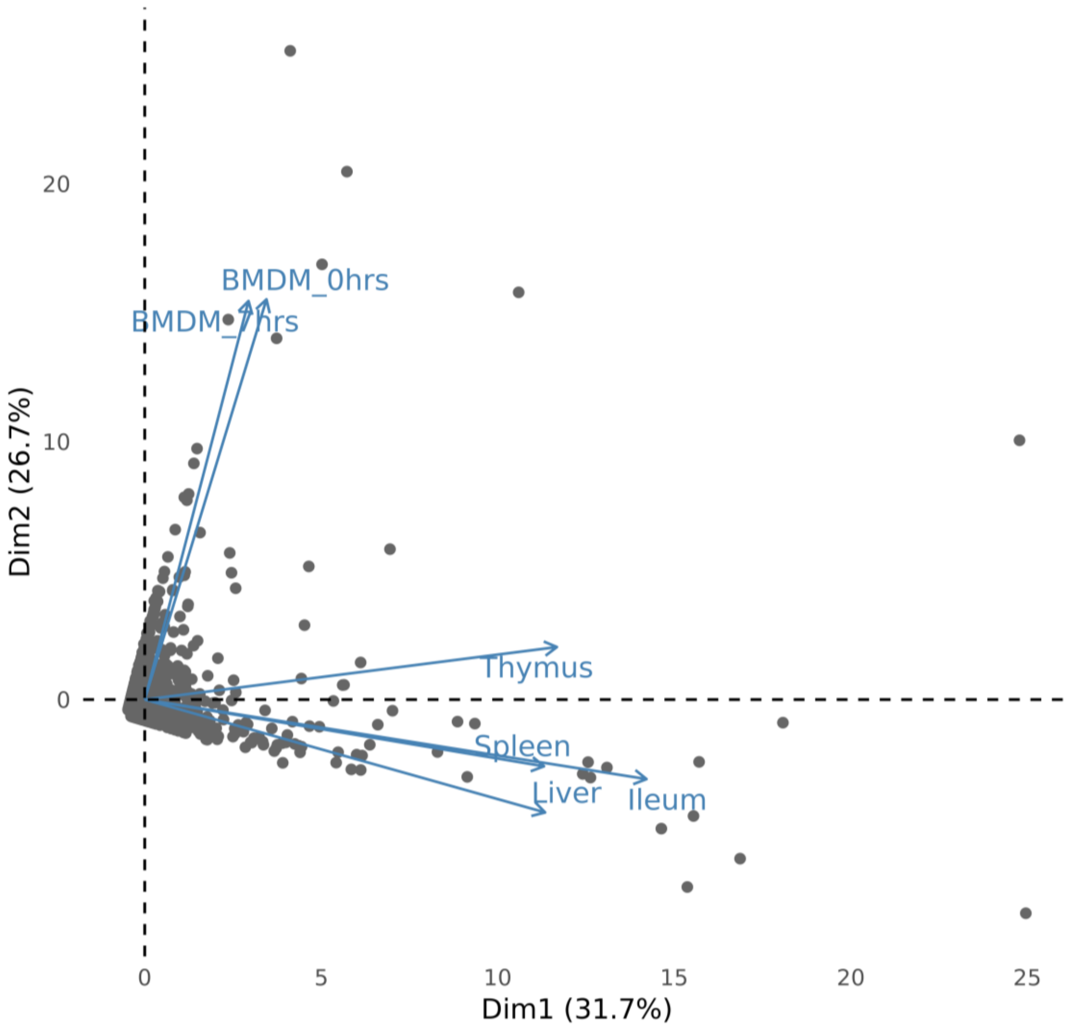
BMDM_0hrs
BMDM_7hrs
Thymus
Spleen
Liver
Ileum
Dim2 (26.7%)
Dim1 (31.7%)
20
10
0
0
5
10
15
20
25

C

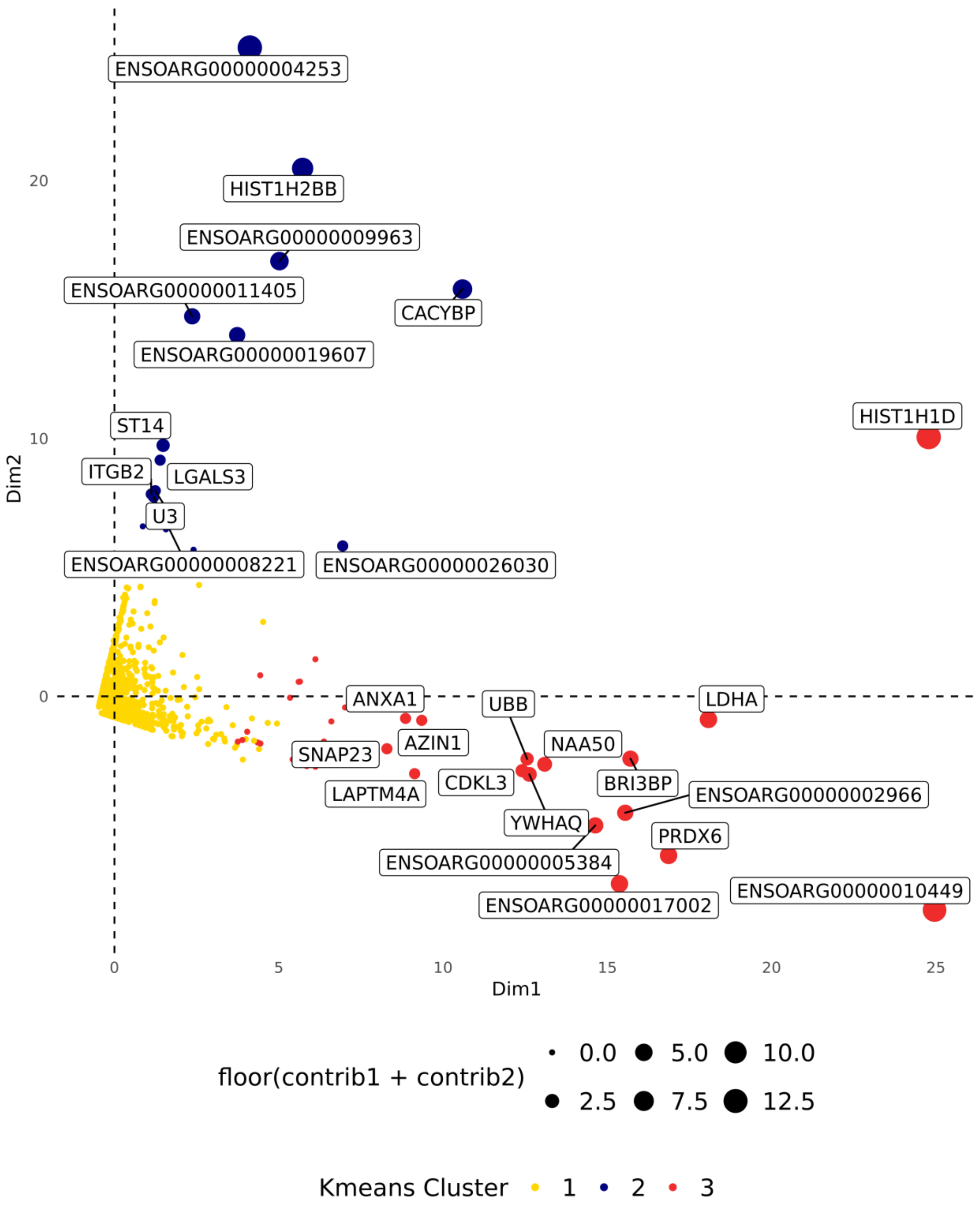

Figure S9. Principle component analysis and Kmeans clustering of ASE gene profiles of 4 tissues and 1 cell type (2 treatment) in 6 sheep. The gene list comprised of all ASE genes in each tissue or cell type was used to perform PCA which established the presence of a hierarchical stratification resulting in 3 groups in the dataset. This grouping was used to cluster the dataset using K means algorithm with 3 centres. **A)** Samples PCA plot of the first 2 PCs (Matrix 6 sample rows x 3000 gene columns) **B)** Genes PCA plot of the first 2 PC (Matrix 3000 gene rows x 6 sample columns) **C)** Kmeans Clusters overlay on the PC1

vs PC2 scatter plot from section B (Genes PCA plot). Gene name labels were included only for the genes with overall contribution to the first 2 components > 1.

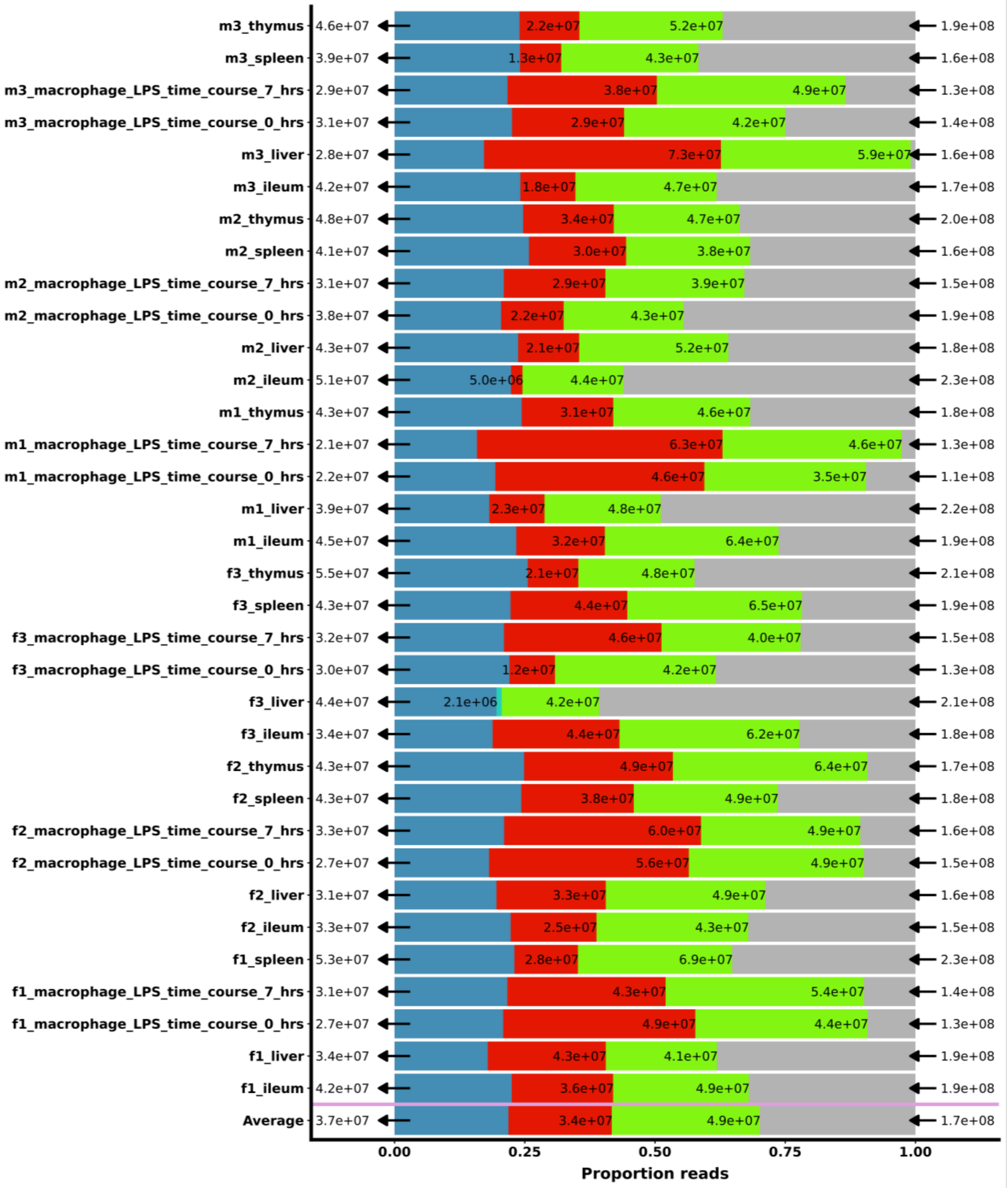

Figure S10. The read loss proportion during the WASP reference mapping bias removal steps. The graph shows the input (read counts on the right) and output (read counts on the 2nd left) RNA-Seq reads from the study before and after WASP processing respectively. Each bar represents 4 overlaid read proportions labelled by colour and raw read count. The total portion of reads coloured in grey shows 100% of the reads processed by WASP (input 170 million reads on average). The green portion shows the non-intersecting reads (and their duplicates) mapped within the range of Ensemble gene coordinate boundaries (exonic, intronic and UTRs

reads 49 million reads on average). The red portion of the reads represents reads intersecting bi-allelic heterozygote sites and selected for synthetic read production step (34 million reads on average). The blue portion of the graph represents the final output of WASP's pipeline (reference biased and duplicated reads removed from red and green portions respectively). The output portion (37 million reads on average) was used in GATK ASEReadCounter for allelic read counts. The last row on the bottom shows the average of these proportions across both tissue and cell type samples. Reference biased reads count for average 20% of input RNA-Seq data.

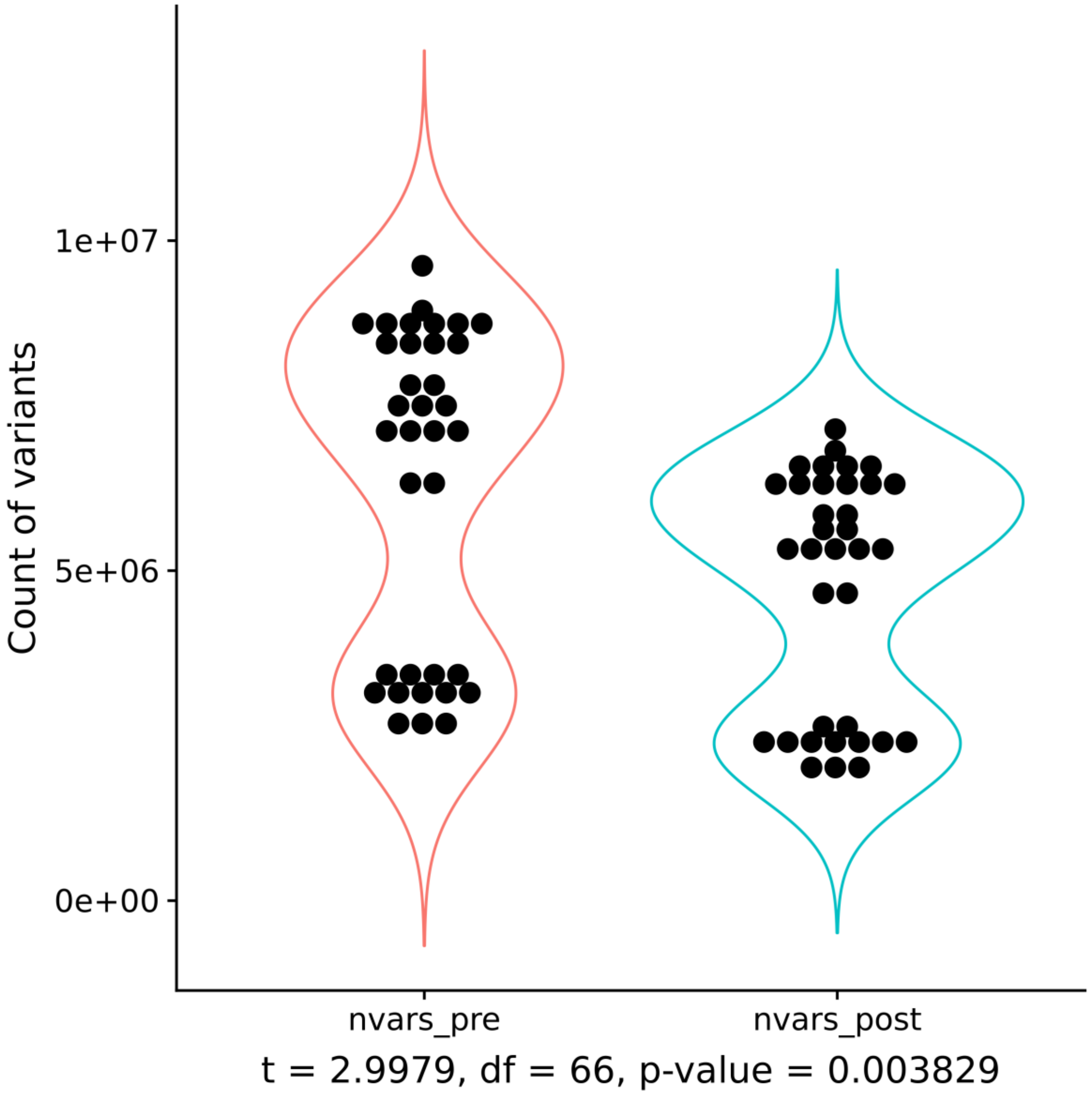

Figure S11. Number of SNVs before and after WASP reference bias correction. There is a significant reduction in number of variants captured (25% on average 1.5e+07) between two steps. Tissues samples dots mainly present within the upper quantile and BMDMs in the lower. Average number of variants beforehand = 6,325,570 and afterwards = 4,729,639.

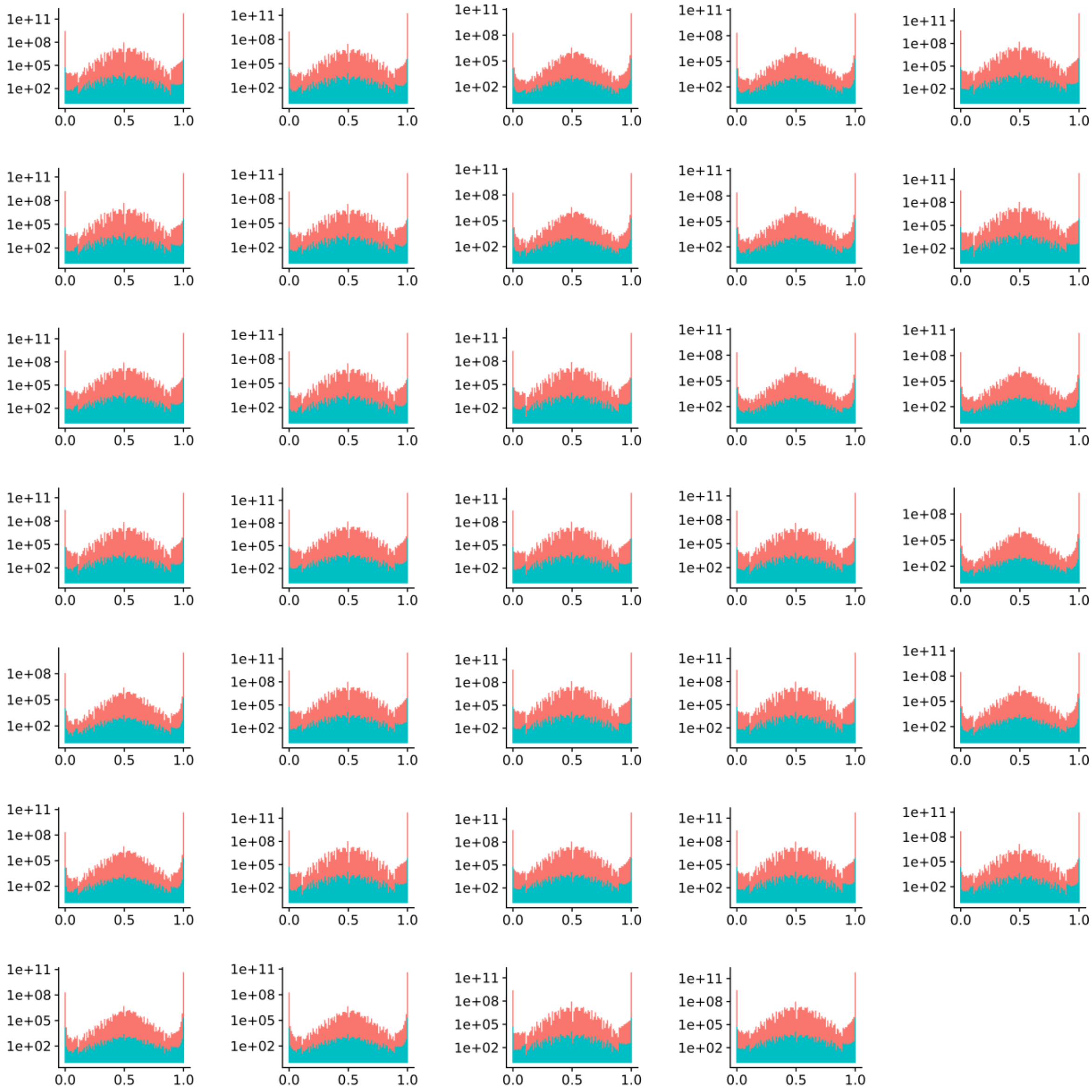

Figure S12. The comparison of reference allele count ratio before and after WASP mapping bias correction .The distribution of refRatio (refCount/refCount+altcounts) in all the tissue and cell samples before (in red) and after WASP (in green). The overall counts , left skew (*viz.* range 0.75-1) and refRatio=1 inflation is significantly reduced after WASP bias correction in all samples.

Table S1. Mean allelic imbalance (log2(ASE_{+LPS}/ASE_{-LPS})) in all 6 sheep. Only 1 gene (VCAN including 7 SNVs) exhibited inducible ASE which was shared between 3 sheep (in bold), while two (GCNT1 and SYNE3) showed inducible ASE in two sheep. Diverse and highly individual ASE response to LPS was observed in each sheep.

Gene	Female1	Female2	Female3	Male1	Male2	Male3
ACOD1	-	-	-	6.8	-	-
ASH2L	-	-	-	-	3.9	-

CASP8	-	-	-	-	-	9.8
CCDC80	-	-	9.1	-	-	-
CLDN1	-	-	-	-	-	10.3
CPOX	-	-	-	-	-	6.5
EDNRB	-	-	-	-	-	11.4
EMP1	-	-	-	-	-	23.9
ENSOARG00000001174	-	-	-	-	5.0	-
ENSOARG00000001910	-	-	-	-	-	7.8
ENSOARG00000002108	-	-	-	-	-	8.0
ENSOARG00000002898	-	-	-	-	-	5.3
ENSOARG00000004106	-	-	-	-	-	7.5
ENSOARG00000006889	-	-	31.9	-	-	-
ENSOARG00000007547	-	-	-	-	8.2	-
ENSOARG00000007603	-	-	-	6.9	-	-
ENSOARG00000014875	-	-	-	-	-	5.6
ENSOARG00000016081	6.4	-	-	-	-	-
ENSOARG00000018177	-	-	11.0	-	-	-
ENSOARG00000021562	-	-	-	-	-	9.0
FDX1	-	-	-	-	17.3	-
GCNT1	-	-	-	16.9	-	30.4
GSDME	-	-	-	4.6	-	-
HNMT	-	-	-	-	-	15.1
HUNK	-	-	-	-	-	4.0
IL1RN	-	-	-	-	-	9.7
KANSL1L	-	-	-	-	-	4.7
KMO	-	-	-	-	-	9.7
MB21D2	-	-	-	5.3	-	-
MYO10	-	-	-	-	-	7.8
NRP1	-	-	9.3	-	-	-
OGFRL1	-	-	-	-	-	16.5
PDE4D	-	-	-	-	-	11.7
PDK4	-	-	-	6.9	-	-
PGM2	-	-	-	-	-	9.5
PLK2	-	-	-	-	-	6.7
PPIF	6.9	-	-	-	-	-
PRNP	-	-	-	-	-	8.8
RASSF3	-	-	-	-	9.2	-
RGS1	-	-	-	-	-	8.4
SAFB2	-	-	-	-	-	5.3
SIGLEC15	-	-	-	4.8	-	-
SLC35F2	-	-	-	-	6.5	-
SYDE1	-	-	-	-	-	5.1
SYNE3	-	-	7.2	-	-	10.7
TBC1D1	-	-	-	6.3	-	-
TFEC	-	-	10.3	-	-	-
TRAF1	12.1	-	-	-	-	-

VCAN	23.5	-	-	-	14.0	13.9
VLDLR	-	-	-	-	4.7	-
